# The Contributions of Wobbling and Superwobbling to the Reading of the Genetic Code

**DOI:** 10.1371/journal.pgen.1003076

**Published:** 2012-11-15

**Authors:** Sibah Alkatib, Lars B. Scharff, Marcelo Rogalski, Tobias T. Fleischmann, Annemarie Matthes, Stefanie Seeger, Mark A. Schöttler, Stephanie Ruf, Ralph Bock

**Affiliations:** Max-Planck-Institut für Molekulare Pflanzenphysiologie, Potsdam-Golm, Germany; National Institutes of Health, United States of America

## Abstract

Reduced bacterial genomes and most genomes of cell organelles (chloroplasts and mitochondria) do not encode the full set of 32 tRNA species required to read all triplets of the genetic code according to the conventional wobble rules. Superwobbling, in which a single tRNA species that contains a uridine in the wobble position of the anticodon reads an entire four-fold degenerate codon box, has been suggested as a possible mechanism for how tRNA sets can be reduced. However, the general feasibility of superwobbling and its efficiency in the various codon boxes have remained unknown. Here we report a complete experimental assessment of the decoding rules in a typical prokaryotic genetic system, the plastid genome. By constructing a large set of transplastomic knock-out mutants for pairs of isoaccepting tRNA species, we show that superwobbling occurs in all codon boxes where it is theoretically possible. Phenotypic characterization of the transplastomic mutant plants revealed that the efficiency of superwobbling varies in a codon box-dependent manner, but—contrary to previous suggestions—it is independent of the number of hydrogen bonds engaged in codon-anticodon interaction. Finally, our data provide experimental evidence of the minimum tRNA set comprising 25 tRNA species, a number lower than previously suggested. Our results demonstrate that all triplets with pyrimidines in third codon position are dually decoded: by a tRNA species utilizing standard base pairing or wobbling and by a second tRNA species employing superwobbling. This has important implications for the interpretation of the genetic code and will aid the construction of synthetic genomes with a minimum-size translational apparatus.

## Author Summary

Reduced genomes of parasitic bacteria, chloroplasts, and mitochondria do not encode the full set of 32 tRNAs required to read all triplets of the genetic code according to Francis Crick's wobble rules. tRNAs with U in the wobble position of their anticodon might be able to make up for the deficit by pairing with any of the four bases at the third position of the codon via a mechanism called superwobbling. We have investigated the feasibility of superwobbling in the chloroplast genome of tobacco plants. We find that superwobbling occurs in all codon families where it is theoretically possible, demonstrating that all triplets with pyrimidines in third codon position are dually decoded: by a tRNA utilizing standard base pairing or wobbling and by a second tRNA employing superwobbling. We also show that the efficiency of superwobbling is variable in different codon families. Finally, our data reveal that the minimum number of tRNAs required to sustain protein biosynthesis is 25.

## Introduction

32 tRNA species are needed to read all triplets of the genetic code according to the wobble rules proposed by Francis Crick [1]. However, reduced genomes, such as those of cell organelles (plastids and mitochondria) and some parasitic bacteria (e. g., mycoplasmas), contain fewer tRNA genes than this minimal set [2]. In mitochondria of plants and of some lineages of protozoa, at least some of the missing tRNA species are imported from the cytosol [3], [4] and the possibility of tRNA import from the cytosol has also been suggested for plastids of parasitic plants [5], [6], [7]. However, tRNA import is unlikely to account for the seemingly incomplete tRNA sets in human mitochondria (encoding only 22 tRNA species), plastids (encoding 30 tRNA species) and parasitic bacteria [8], [9], [10]. In these systems, extended wobbling is believed to facilitate translation with a reduced set of tRNA species [9]. Extended wobbling refers to the ability of a single tRNA species to read all four triplets in a codon family. For example, uridine 5-oxyacetic acid at the wobble position enables a single tRNA to read all four triplets in a four-fold degenerate codon box [11]. Extended wobbling is also possible with an unmodified uridine in the wobble position of the anticodon and is also referred to as “four-way wobbling”, “hyperwobbling” or “superwobbling” [2], [12], [13], [14]. Both theoretical considerations [1] and experimental data [10] support the idea that uridine in the wobble position of the anticodon can also engage in base-pairing interactions with U or C in the third codon position and, in this way promote reading of all four triplets in a codon family.

An alternative model of extended wobbling, referred to as the “two-out-of-three” reading hypothesis, was suggested by Lagerkvist [15], [16]. This model defines “strong codons” as triplets with six hydrogen bonds formed by the first two bases of the codon in complementary base paring with the anticodon. In contrast, the first two bases of “mixed codons” have five and the first two bases of “weak codons” have four hydrogen bonds participating in base pairing [16]. The “two-out-of-three” reading hypothesis proposes that “strong codons” (with only G-C interactions between the first two bases of the anticodon and the first two bases of the codon) can be read by relying on base pairing with the first two bases of the anticodon, without a significant contribution of the interaction between the third codon position and the wobble position of the anticodon [15]. Due to their lower number of hydrogen bonds, codon boxes with “mixed codons” would be less likely to be readable by a single tRNA species [16]. In contrast, if the U in the wobble position contributes to the stability of the codon-anticodon interaction, as implied by the superwobble hypothesis, boxes with “mixed codons” may be readable with similar efficiency as “strong codons”.

We have previously shown that the unmodified uridine in the wobble position of the plastid tRNA-Gly(UCC) allows decoding of all four glycine codons (GGA, GGC, GGG and GGU; [10]). Glycine codons are strong codons according to the “two-out-of-three” reading hypothesis. Here we have tested whether codon families of four mixed codons can be read by a single tRNA species. By systematically testing all tRNA species involved in reading four-fold degenerate codon boxes, we have established the complete set of decoding rules for the genetic system of the chloroplast. Moreover, we find that the efficiency of superwobbling varies in different codon boxes and is not directly correlated to the number of hydrogen bonds participating in the codon-anticodon interaction.

## Results

### Superwobbling in the ACN family of mixed codons

To examine the possibility of superwobbling in mixed codons, we investigated the pair of threonine tRNAs encoded in the plastid (chloroplast) genome of higher plants. According to the conventional wobble rules [1], the tRNA-Thr(GGU) encoded by the plastid *trnT-GGU* gene should decode the two threonine triplets with a pyrimidine in third codon position (ACC and ACU), whereas the tRNA-Thr(UGU) encoded by the *trnT-UGU* gene should read the two threonine triplets with a purine in third codon position (ACA and ACG). The tRNA-Thr(UGU) has an unmodified uridine in the wobble position of the anticodon in all organelles and bacterial species with reduced tRNA sets (mycoplasmas), where its sequence was determined (http://trnadb.bioinf.uni-leipzig.de/). The assumption that the tRNA-Thr(UGU) can superwobble, but the tRNA-Thr(GGU) cannot (because, for steric reasons, the purines A and G in third codon position should not be capable of base pairing with the purine base guanine in the wobble position of the anticodon), would lead to the following testable predictions: (i) the *trnT-GGU* gene should be non-essential, (ii) the *trnT-UGU* should be essential, and (iii) the tRNA-Thr(UGU) alone should be sufficient to sustain plastid translation, at least to some extent.

**Figure 1:**
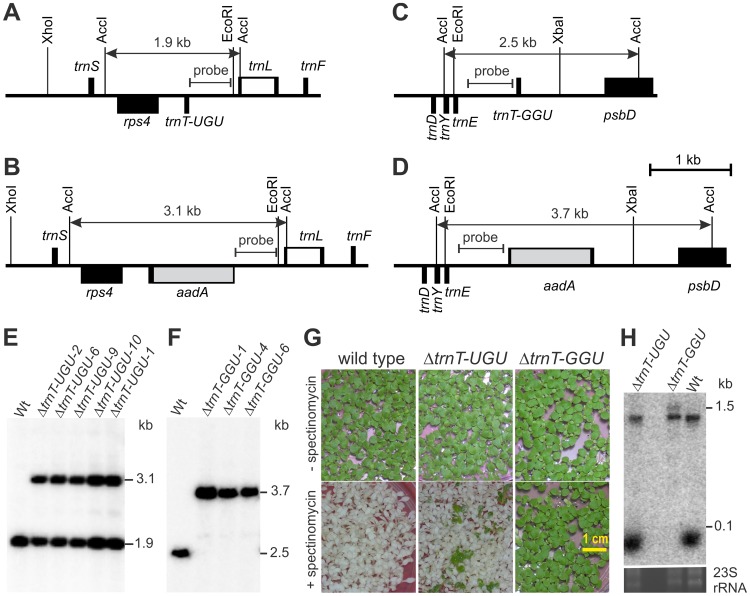
Targeted inactivation of the two plastid *trnT* genes. (A) Physical map of the *trnT-UGU* containing region in the tobacco plastid genome (ptDNA; [44]). Genes above the line are transcribed from the left to the right, genes below the line are transcribed in the opposite direction. Selected restriction sites used for cloning or RFLP analysis are indicated. The hybridization probe and the expected sizes of detected DNA fragments are also shown. Introns are represented by open boxes. (B) Map of the transformed plastid genome in *ΔtrnT-UGU* transplastomic plants. The selectable marker gene *aadA* (grey box) is inserted into the *trnT-UGU* gene in the same transcriptional orientation. (C) Physical map of the *trnT-GGU* containing region in the tobacco ptDNA. (D) Map of the transformed plastid genome in Δ*trnT-GGU* plants. (E) RFLP analysis of Δ*trnT-UGU* transplastomic lines. Independently generated transplastomic lines are designated by Arabic numerals following the tRNA gene name. All transplastomic lines remain heteroplasmic and show both the 1.9 kb wild type-specific hybridization band and the 3.1 kb band diagnostic of the transformed plastid genome. Wt: wild type. (F) RFLP analysis of Δ*trnT-GGU* transplastomic plants. All lines are homoplasmic and show exclusively the 3.7 kb band diagnostic of the transgenic ptDNA. (G) Seed assays to confirm heteroplasmy of Δ*trnT-UGU* plants and homoplasmy of Δ*trnT-GGU* plants. Seeds were germinated in the absence or in the presence of spectinomycin. Δ*trnT-UGU* plants produce mostly antibiotic-sensitive seedlings and a few antibiotic-resistant seedlings, as expected for a heteroplasmic situation. Moreover, many of the resistant seedlings are variegated indicating their composition of tissues possessing and tissues lacking the transgenic plastid genome. In contrast, the Δ*trnT-GGU* lines produce homogeneous antibiotic-resistant progeny, confirming their homoplasmic status. (H) Analysis of tRNA-Thr(GGU) accumulation in the wild type, a heteroplasmic Δ*trnT-UGU* line and a homoplasmic Δ*trnT-GGU* line by northern blotting. Hybridization of electrophoretically separated RNA isolated from purified chloroplasts to a plastid *trnT-GGU* probe confirms complete lack of mature tRNA-Thr(GGU) in the Δ*trnT-GGU* homoplasmic knock-out line, whereas its accumulation is unaltered in the heteroplasmic Δ*trnT-UGU* line. Note accumulation of a ∼1.5 kb hybridizing RNA species in the Δ*trnT-GGU* line, which corresponds to the tRNA-Thr(GGU) disrupted with the *aadA* cassette. To control for RNA loading, part of the ethidium bromide-stained gel (containing the two largest 23S rRNA hidden break products) prior to blotting is also shown.

To test these predictions, we constructed knock-out alleles for both *trnT* genes in the plastid genome of the model plant tobacco (*Nicotiana tabacum*; Figure 1A–1D). The genes were disrupted by insertion of a selectable marker gene for chloroplast transformation (*aadA*) conferring spectinomycin resistance [17]. The knock-out alleles were then introduced into the plastid genome by particle gun-mediated transformation. Homologous recombination resulted in replacement of the wild-type allele with the knock-out allele. The resulting stably transformed (transplastomic) lines are subsequently referred to as Δ*trnT-UGU* lines and Δ*trnT-GGU* lines, respectively. Targeted disruption of the tRNA genes was confirmed by Southern blot analyses, which produced the expected restriction fragment length polymorphisms (Figure 1E, 1F).

Due to the polyploidy of the plastid genome, the knock-out of an essential gene results in balancing selection for two antagonistic genome types: the wild-type genome (expressing the essential gene but not the antibiotic resistance) and the transformed genome (expression the antibiotic resistance but not the essential gene). Consequently, a stable mix of both genome types (heteroplasmy) is observed under antibiotic selection [18], [19], [20]. This stable heteroplasmy was clearly seen in all Δ*trnT-UGU* lines (Figure 1E), consistent with the prediction that this gene should be essential.

Growth in the absence of antibiotic selection releases the balancing selection in that it abrogates the selective pressure for maintenance of the transgenic plastid genome. This leads to predominant genome segregation towards the wild-type genome, which can be easily visualized by germinating seeds harvested from such plants on antibiotic-containing synthetic medium (where seedlings that lack the transgenic plastid genome bleach out; [21], [22]). This was observed in all Δ*trnT-UGU* lines (Figure 1G), providing further evidence of the essentiality of the *trnT-UGU* gene.

In contrast to *trnT-UGU*, the *trnT-GGU* gene turned out to be non-essential. Both DNA gel blot analyses and inheritance assays clearly demonstrated that homoplasmic knock-out plants had been obtained which lack residual wild-type copies of the plastid genome (Figure 1F, 1G). In addition, RNA gel blot analyses confirmed complete absence of tRNA-Thr(GGU) molecules from the Δ*trnT-GGU* tobacco lines (Figure 1H), strongly suggesting that plastid translation can proceed in the absence of this tRNA species (and making it unlikely that this tRNA is imported from the cytosol). Thus, the tRNA-Thr(UGU) is necessary and sufficient to sustain translation, indicating that superwobbling in mixed codons is possible.

### The single plastid alanine tRNA is essential

The plastid alanine tRNA-Ala(UGC) may exemplify obligatory superwobbling in that there is only a single alanine tRNA species encoded in the chloroplast genome and it has a uridine in the wobble position of the anticodon. The U remains unmodified in mitochondria of the fungus *Neurospora crassa* as well as in the reduced bacterial genomes of the genus Mycoplasma (http://trnadb.bioinf.uni-leipzig.de/), but may carry an unknown modification in plastids [23]. Given the essentiality of plastid translation [19], [24] and the probable absence of tRNA import into plastids [8], [10], [22], this single alanine tRNA species is predicted to be encoded by an essential gene. To test this assumption, we constructed a knock-out allele for the *trnA(UGC)* gene and introduced it into the tobacco plastid genome (Figure S1). Molecular analysis of the generated Δ*trnA-UGC* knock-out lines revealed stable heteroplasmy of the plastid genome under antibiotic selection (Figure S1D). Moreover, upon growth in soil, the heteroplastomic transplastomic plants displayed the characteristic leaf-loss phenotype (Figure S1F) caused by segregation into homoplasmy for the knock-out of an essential gene. This leads to cessation of cell division and ultimately to cell death, which in turn produces misshapen leaves that lack parts of their blade [20], [22], [24]. Finally, the Δ*trnA-UGC* knock-out lines also showed a strong tendency to lose the transformed plastid genome in the absence of selective pressure, as evidenced by seed assays (Figure S1E). Taken together, these data suggest that the single plastid genome-encoded alanine tRNA is essential for translation and its loss cannot be complemented by tRNA import from the cytosol.

### Superwobbling occurs in all four-fold degenerate codon boxes

To obtain a complete picture of the decoding rules in plastids and to determine the minimum tRNA set that is necessary and sufficient to facilitate translation, we next wanted to examine all cases in which wobbling and/or superwobbling theoretically allow a reduction of tRNA species. In addition to the GGN glycine box [10] and the ACN threonine box, this concerns two other four-fold degenerate boxes of mixed codons: the UCN serine box and the GUN valine box. For each of these boxes, two tRNA genes are present in the plastid genome and, if superwobbling occurs, the tRNA species with a U in the wobble position of the anticodon should suffice. Thus, the *trnS-UGA* and *trnV-UAC* genes should be essential and, if they are capable of superwobbling, the *trnS-GGA* and *trnV-GAC* genes should be non-essential. In addition to the UCN box, serine also has two codons in the AGN box (AGC and AGU). These triplets are most likely exclusively served by a third serine tRNA species, tRNA-Ser(GCU), and, therefore, the plastid gene encoding this tRNA species is also expected to be essential.

**Figure 2:**
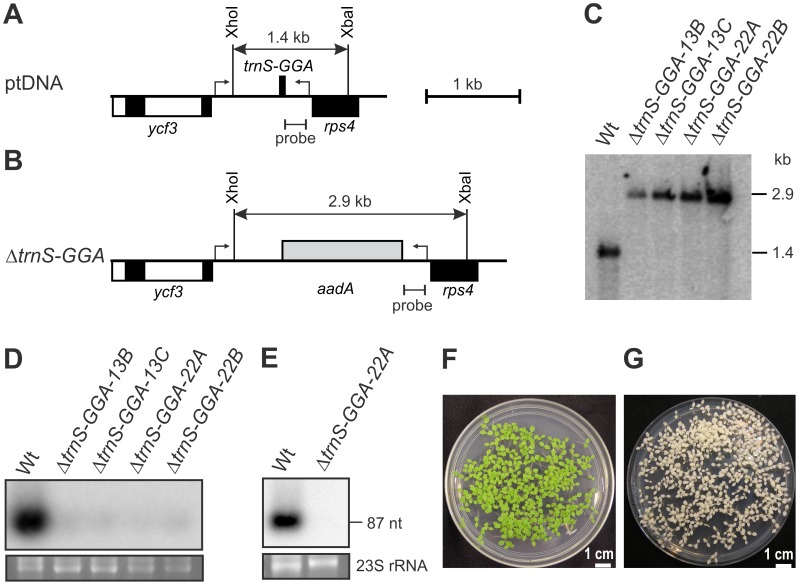
Targeted deletion of the plastid *trnS-GGA* gene. (A) Physical map of the region in the tobacco plastid genome harboring the *trnS-GGA* gene. Genes above the line are transcribed from the left to the right, genes below the line are transcribed in the opposite direction. The bent arrows indicate the borders of the transformation plasmid. Restriction sites used for RFLP analysis are indicated. The hybridization probe and the expected sizes of detected DNA fragments are also shown. Introns are represented by open boxes. (B) Map of the transformed plastid genome in Δ*trnS-GGA* transplastomic lines. The *aadA* cassette is shown as grey box. (C) RFLP analysis of Δ*trnS-GGA* plastid transformants. All lines are homoplasmic and show exclusively the 2.9 kb band diagnostic of the transplastome. Independently generated transplastomic lines are designated by Arabic numerals following the tRNA gene name, the following capital letter indicates an individual plant. Wt: wild type. (D) tRNA-Ser(GGA) accumulation in wild-type plants and Δ*trnS-GGA* transplastomic lines. Hybridization to a plastid *trnS-GGA* probe reveals weak signals in all transplastomic plants, which are presumably caused by cross-hybridization to the mitochondrial *trnS-GGA*. To control for RNA loading, part of the ethidium bromide-stained gel (showing the two largest 23S rRNA hidden break products) prior to blotting is also shown. (E) tRNA-Ser(GGA) accumulation in isolated chloroplasts of wild type plants and a Δ*trnS-GGA* knock-out line. Hybridization to the plastid *trnS-GGA* probe confirms complete absence of the tRNA from the transplastomic line. (F) Confirmation of homoplasmy of the Δ*trnS-GGA* lines by inheritance assays. Germination of seeds harvested from transplastomic plants on spectinomycin-containing medium results in a homogeneous population of green antibiotic-resistant seedlings. (G) Comparison with spectinomycin-sensitive wild-type seedlings. Antibiotic sensitivity is evidenced by the white phenotype of all seedlings.

**Figure 3:**
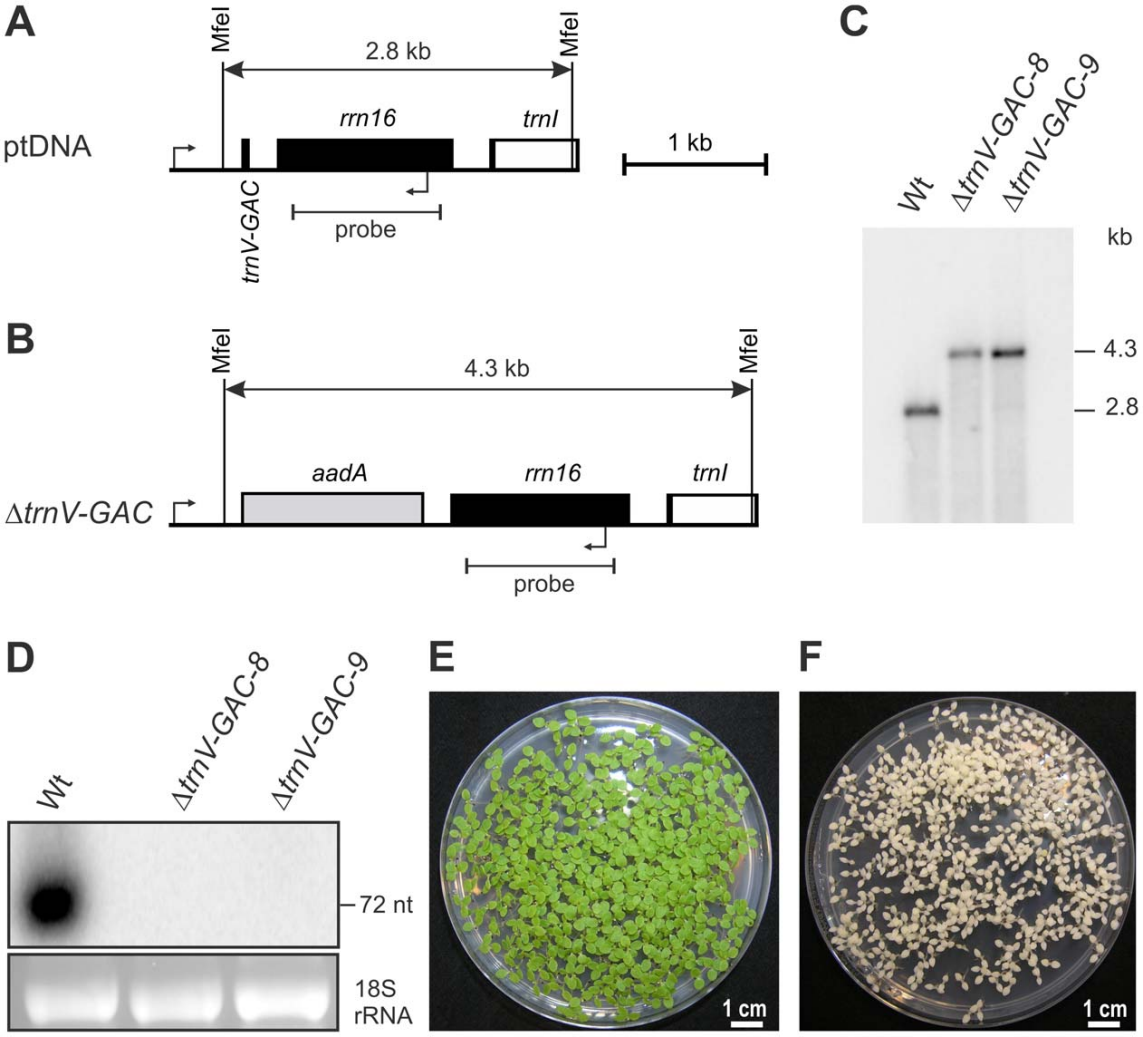
Targeted deletion of the plastid *trnV-GAC* gene. (A) Physical map of the region in the tobacco plastid genome containing *trnV-GAC*. All genes shown transcribed from the left to the right. The bent arrows indicate the borders of the transformation plasmid. Restriction sites used for RFLP analysis are indicated. The hybridization probe and the expected sizes of detected DNA fragments are also shown. Introns are represented by open boxes. (B) Map of the transformed plastid genome in Δ*trnV-GAC* lines. The *aadA* cassette is shown as grey box. (C) RFLP analysis of Δ*trnV-GAC* plastid transformants. Wt: wild type. (D) tRNA-Val(GAC) accumulation in the wild type and in Δ*trnV-GAC* lines determined by northern blotting. Hybridization to a plastid *trnV-GAC* probe confirms complete absence of the tRNA from homoplasmic transplastomic lines. To control for RNA loading, the 18S rRNA-containing part of the ethidium bromide-stained agarose gel prior to blotting is also shown. (E) Homoplasmy of Δ*trnV-GAC* lines as confirmed by inheritance assays. Transplastomic seeds germinated on spectinomycin-containing yield a homogeneous population of green antibiotic-resistant seedlings. (F) Wild-type seedlings are sensitive to spectinomycin and bleach out in the presence of the antibiotic.

To test for decoding of serine and valine triplets by superwobbling, we analyzed the three plastid tRNA-Ser genes and the two tRNA-Val genes for their essentiality in knock-out experiments. Consistent with reading of UCU and UCC serine codons and GUU and GUC valine codons by superwobbling, both the *trnS-GGA* gene and the *trnV-GAC* gene were found to be non-essential (Figure 2 and Figure 3). The knock-out mutants were homoplasmic in Southern blot analyses (Figure 2C and Figure 3C), showed no detectable accumulation of the knocked-out tRNA species (Figure 2D, 2E and Figure 3D) and the seeds germinated as a homogeneous population of spectinomycin-resistant seedlings (Figure 2F, 2G and Figure 3E, 3F). Non-essentiality of the tRNA-Val(GAC) is in agreement with the existence of a deletion mutant in the plastid genome that comprises the *trnV-GAC* gene [25].

In contrast, the *trnS-UGA*, *trnS-GCU* and *trnV-UAC* genes turned out to be essential (Figures S2, S3, S4). All mutant plants remained heteroplasmic, showed the typical leaf-loss phenotype upon growth in soil and only rarely transmitted the transformed plastid genome into the next generation (Figures S2, S3, S4).

Together, these results demonstrate that all four-fold degenerate codon boxes can be read by a single tRNA species. With a single exception (see analysis of the arginine tRNAs below), this tRNA species is always the one with uridine in the wobble position and, in all cases, is encoded by an essential gene. Consequently, the second tRNA species that, according to the classic wobble rules, should read the two triplets with a pyrimidine nucleotide in third codon position, is always non-essential.

### Wobbling makes the *trnL-CAA* gene dispensable

Organellar genomes make maximum use of Crick's wobble rules [1] in that usually only a single tRNA species exists for each pair of codons (a pair being either the two triplets of a codon box with a purine in third position or the two triplets with a pyrimidine in third position). In the genomes of higher plant plastids, there is only a single exception: two distinct tRNA species exist for the two leucine codons UUA and UUG. These tRNAs are encoded by the *trnL-UAA* and *trnL-CAA* genes and the wobble positions of their anticodons are modified to 2′-O-methyluridine and 2′-O-methylcytidine, respectively [26]. Whether or not the base methylation in the wobble position enhances the specificity of decoding or perhaps even necessitates two distinct tRNA species for the reading of UUA and UUG triplets, is not known.

**Figure 4:**
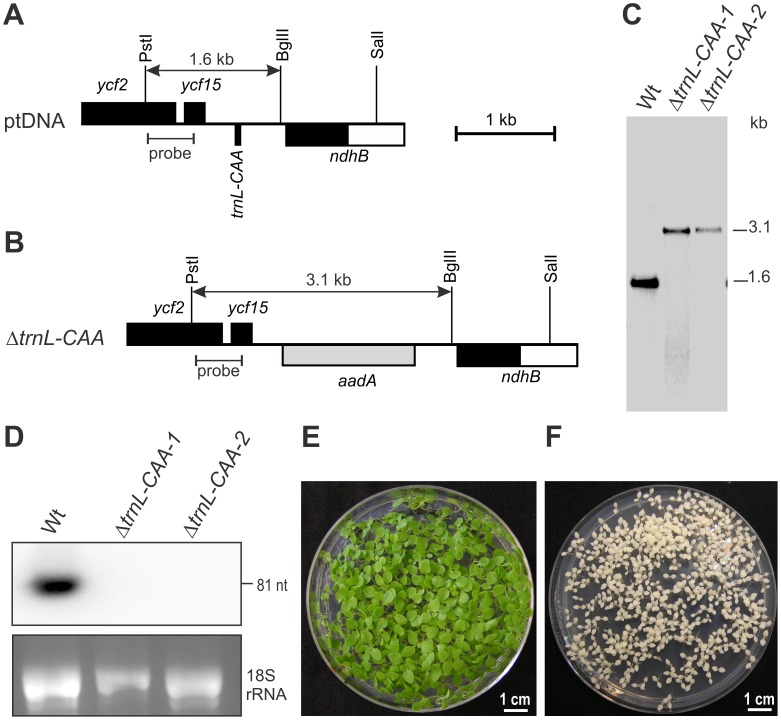
Targeted deletion of the plastid *trnL-CAA* gene. (A) Physical map of the region in the tobacco plastid genome containing the gene for *trnL-CAA*. Genes above the line are transcribed from the left to the right, genes below the line are transcribed in the opposite direction. Selected restriction sites used for cloning and RFLP analysis are indicated. The hybridization probe and the expected sizes of detected DNA fragments are also shown. Introns are represented by open boxes. (B) Map of the transformed plastid genome in Δ*trnL-CAA* transplastomic plants. The *aadA* cassette replacing *trnL-CAA* is shown as grey box. (C) RFLP analysis of Δ*trnL-CAA* plastid transformants. All lines are homoplasmic and show exclusively the 3.1-kb band diagnostic of the transplastome. Wt: wild type. (D) tRNA-Leu(CAA) accumulation in the wild type and Δ*trnL-CAA* lines assessed by northern blotting. Hybridization to a plastid *trnL-CAA* probe confirms complete absence of the tRNA from homoplasmic knock-out lines. The ethidium bromide-stained agarose gel prior to blotting is also shown. (E) Confirmation of the homoplasmic state of the Δ*trnL-CAA* lines by inheritance assays. Germination of seeds from transplastomic plants on spectinomycin-containing medium results in a homogeneous population of green antibiotic-resistant seedlings. (F) Wild-type seedlings are sensitive to spectinomycin and bleach out in the presence of the antibiotic.

To address these questions, we generated knock-out tobacco plants for the plastid *trnL-UAA* and *trnL-CAA* genes (Figure 4 and Figure S5). While homoplasmic knock-out lines were readily obtained for the *trnL-CAA* gene (Figure 4), the transplastomic Δ*trnL-UAA* plants remained heteroplasmic and also fulfilled all other criteria of a mutant for an essential plastid gene (Figure S5). This indicates that the tRNA-Leu(UAA) can read both UUA and UUG triplets and suggests that 2′-O-methyluridine can wobble with G in the third codon position.

The four other leucine codons (in the CUN box) are most probably read by the third leucine tRNA species which is encoded by the plastid *trnL-UAG* gene. The tRNA-Leu(UAG) has an unmodified U in the wobble position (but an m^7^G modification in position 36 of the anticodon loop; [26], [23]). In the absence of a tRNA species with a GAG anticodon, the tRNA-Leu(UAG) should be capable of reading CUU and CUC triplets by superwobbling. As expected, transplastomic knock-out experiments confirmed essentiality of the *trnL-UAG* gene (Figure S6).

Previous work has demonstrated that 7-methylguanosine at the wobble position allows base pairing with all four nucleotides, A, C, U and G [27]. In tRNA-Leu(UAG), the m^7^G modification is not in the wobble position, but in position 36, which base pairs with the first codon position. This raises the theoretical possibility that pairing of m^7^G with both C and U in first codon position could allow not only reading of CUN codons, but also of the two leucine codons in the UUN box, UUA and UUG. However, essentiality of the Δ*trnL-UAA* (Figure S5) excludes this possibility and demonstrates that tRNA-Leu(UAG) cannot decode UUA and UUG triplets.

### The two arginine tRNAs are essential

The plastid genome encodes two tRNAs for arginine, tRNA-Arg(UCU) and tRNA-Arg(ACG). The tRNA-Arg(UCU) should decode AGA and AGG triplets, whereas the four arginine triplets in the CGN box should be read by tRNA-Arg(ACG). The tRNA-Arg(ACG) carries an adenosine-to-inosine modification in the wobble position, which is thought to enable reading of all four nucleotides in the third position of CGN codons (possibly by “two-out-of-three” reading; [28], [29]). Considering the presence of only two tRNA species for the six arginine codons and the properties of their anticodons, there should be no possibility for further tRNA reduction by employing superwobbling. To confirm this assumption, we analyzed the two genes for plastid arginine tRNAs, *trnR-ACG* and *trnR-UCU*, by reverse genetics (Figures S7 and S8). Molecular, and phenotypic analyses as well as inheritance assays revealed heteroplasmy of all transplastomic lines (Figures S7 and S8), thus suggesting essentiality of both arginine tRNA genes.

### Anticodon-dependent efficiency of wobbling and superwobbling

**Figure 5:**
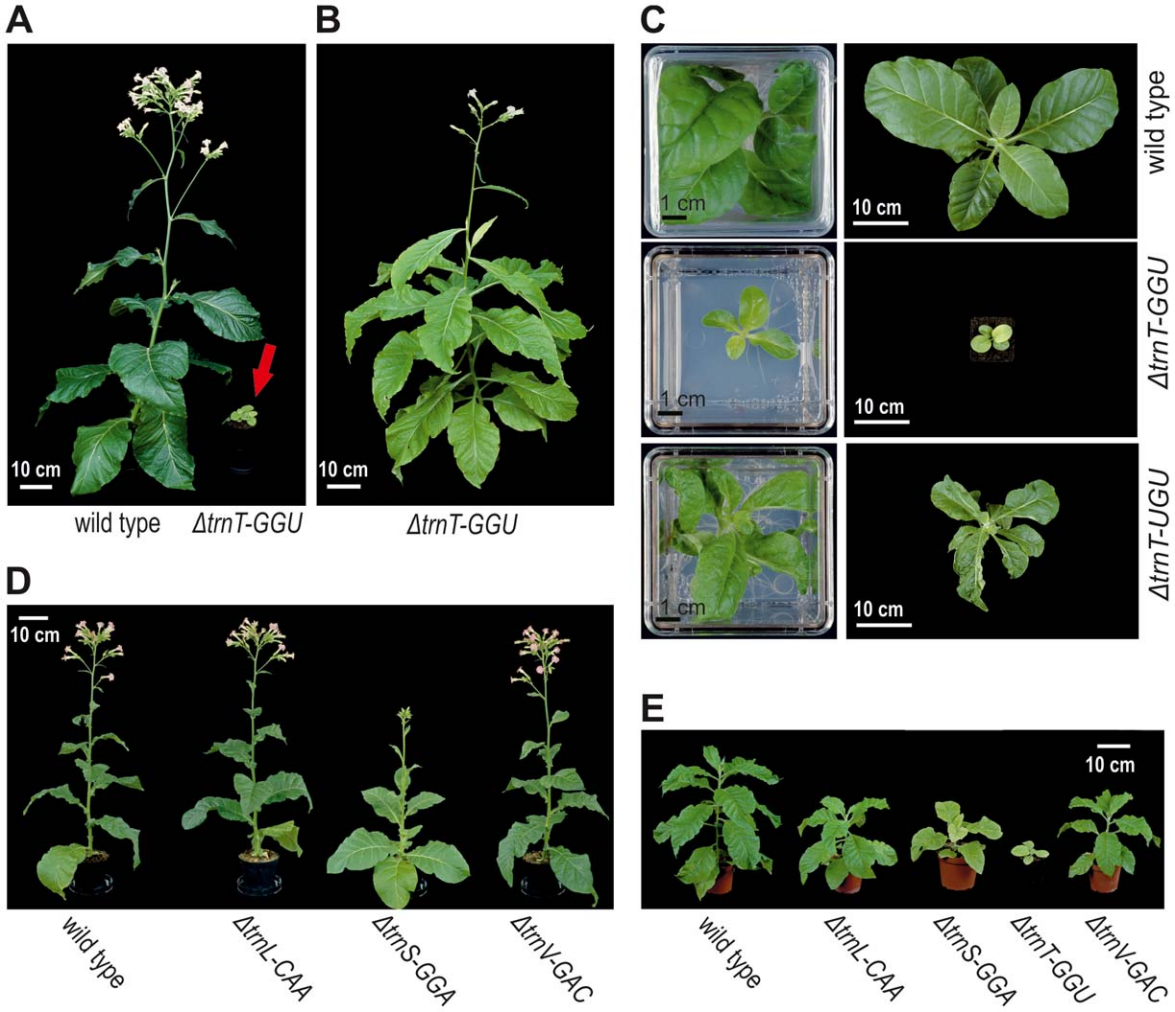
Phenotypes of transplastomic plants generated with knock-out constructs for the tRNA genes *trnT-UGU*, *trnT-GGU*, *trnL-CAA*, *trnS-GGA*, and *trnV-GAC*. (A) Growth phenotype of a Δ*trnT-GGU* plant in comparison with a wild-type plant. Plants were grow from seeds in soil (with nitrogen-rich fertilizer) under ∼100 µE m^−2^ s^−1^ light intensity and photographed after 12 weeks. The red arrow points to the Δ*trnT-GGU* plant. (B) Flowering and seed set of a Δ*trnT-GGU* plant after 50 weeks of growth under ∼20 µE m^−2^ s^−1^ light intensity. (C) Growth of Δ*trnT-GGU* and Δ*trnT-UGU* plants in comparison with a wild-type plant after 6 weeks of growth on sucrose-containing synthetic medium (left pictures) and a subsequent 16 day growth period in soil (right pictures). Note the typical leaf-loss phenotype in the Δ*trnT-UGU* plant indicating essentiality of the tRNA gene [19], [20]. (D) Phenotypes of Δ*trnL-CAA*, Δ*trnS-GGA* and Δ*trnV-GAC* transplastomic plants in comparison with a wild-type plant after growth for 13 weeks in soil under standard greenhouse conditions (average light intensity: 200 µE m^−2^ s^−1^). (E) Phenotype of Δ*trnL-CAA*, Δ*trnS-GGA*, Δ*trnT-GGU* and Δ*trnV-GAC* transplastomic plants in comparison with a wild-type plant after growth for 20 weeks under low-light conditions (∼80 µE m^−2^ s^−1^).

In the course of this work, we identified four non-essential plastid tRNA genes: *trnT-GGU*, *trnL-CAA*, *trnS-GGA* and *trnV-GAC*. To assess the phenotypic consequences of the lack of these tRNAs, we compared transplastomic mutant plants with wild-type plants under a variety of growth conditions. The Δ*trnT-GGU* plants, initially generated to test whether or not reading of an entire four-fold degenerate codon box by a single tRNA species is also possible in boxes with “mixed codons”, displayed a strong mutant phenotype characterized by reduced leaf pigmentation and severely retarded growth (Figure 5A–5C). This is consistent with the conclusion drawn from a *trnG-GCC* knock-out that superwobbling is less efficient than classic wobbling [10]. Interestingly, while Δ*trnG-GCC* seedlings were nearly white and showed strongly delayed greening both in sterile culture and in soil [10], Δ*trnT-GGU* seedlings were light-green in both synthetic medium and soil (Figure 5A–5C) indicating that, at least under these conditions, superwobbling in mixed ACN codons is no less efficient than superwobbling in strong GGN codons.

The other three homoplasmic tRNA knock-out mutants, Δ*trnL-CAA*, Δ*trnS-GGA* and Δ*trnV-GAC* had much less severe phenotypes. The Δ*trnS-GGA* plants showed a pronounced light-green phenotype, but still grew relatively well under photoautotrophic conditions, in both standard light and low light (Figure 5D, 5E). In contrast, the Δ*trnL-CAA* and Δ*trnV-GAC* plants displayed no discernible phenotype under standard light conditions and showed only a very mild growth retardation under low-light conditions (of ∼80 µE m^−2^ s^−1^; Figure 5D, 5E). The very different growth phenotypes of the Δ*trnT-GGU*, Δ*trnG-GCC*, Δ*trnS-GGA* and Δ*trnV-GAC* knock-out mutants tentatively suggested that the efficiency of superwobbling varies considerably between codon boxes and tRNA isoacceptors, resulting in different efficiencies of plastid translation in the mutants. In agreement with this conclusion, the severity of the phenotypes of the four homoplasmic tRNA knock-out mutants does not correlate with the number of affected codons in the genome (Table S1).

**Figure 6:**
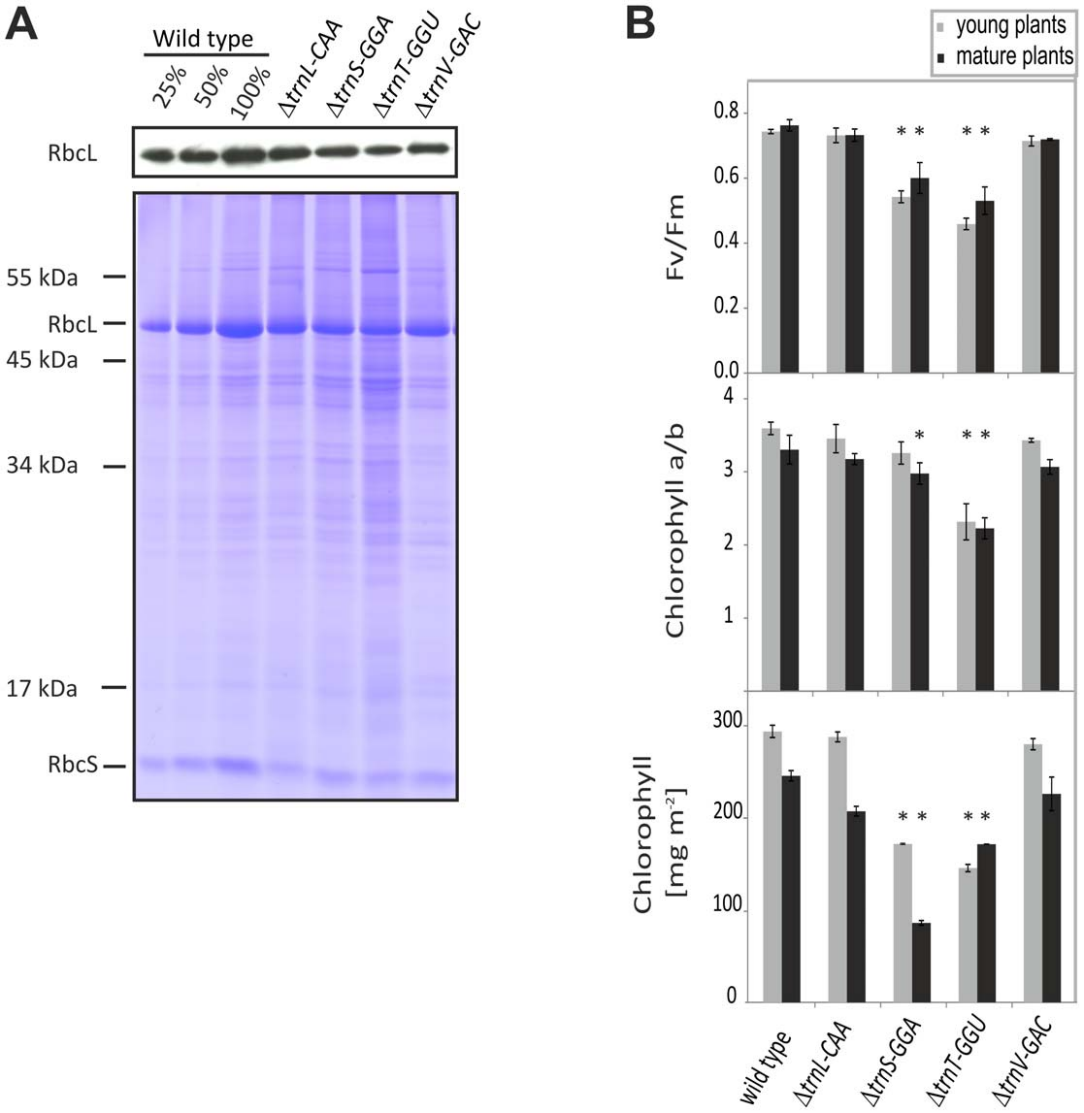
Analysis of plastid protein synthesis and photosynthetic parameters in Δ*trnL-CAA*, Δ*trnS-GGA*, Δ*trnT-GGU*, and Δ*trnV-GAC* plants. (A) Assessment of RbcL protein accumulation by western blotting using a specific anti-RbcL antibody. For semiquantitative analysis, a dilution series of wild-type protein was loaded. Consistent with the differences in the severity of the growth phenotypes (cf. Figure 5), the Δ*trnV-GAC* and Δ*trnL-CAA* mutants show the smallest reduction in RbcL accumulation, whereas the Δ*trnS-GGA* mutant and especially the Δ*trnT-GGU* mutant are more strongly affected, with RbcL levels being in the range of the 25% dilution of the wild-type sample in the Δ*trnT-GGU* mutant. Reduced synthesis of chloroplast proteins is also apparent, when total plant protein samples are separated by gel electrophoresis and stained with Coomassie (lower panel). The two most abundant proteins (representing the large and small subunits of Rubisco, RbcL and RbcS) are indicated. Reduced abundance of chloroplast proteins in the Δ*trnS-GGA* and Δ*trnT-GGU* mutants also becomes evident by a stronger background staining (coming from a large number of lower abundant nuclear-encoded proteins). (B) Analysis of chlorophyll content, chlorophyll a∶b ratio and the maximum quantum efficiency of photosystem II (F_V_/F_M_) in wild-type plants and homoplasmic transplastomic tRNA knock-out mutants. Datasets are shown for plants grown under ∼80 µE m^−2^ s^−1^ light intensity. Young Δ*trnL-CAA*, Δ*trnS-GGA* and Δ*trnV-GAC* plants were measured after 7 weeks of growth, Δ*trnT-GGU* plants after 30 weeks (when they had reached a similar size as the other lines after 7 weeks). Mature Δ*trnL-CAA*, Δ*trnS-GGA* and Δ*trnV-GAC* plants were measured after 20 weeks of growth, Δ*trnT-GGU* plants were raised at ∼20 µE m^−2^ s^−1^ for 40 weeks and then grown for 4 weeks at ∼80 µE m^−2^ s^−1^. The fourth leaf from the top was analyzed. For each plant line, three different plants were measured. F_V_/F_M_ represents the maximum quantum efficiency of PSII in the dark adapted state. The error bars indicate the standard deviation, statistically significant differences from the wild type (p<0.05; Student's t-test) are indicated by asterisks.

To further characterize the effects of superwobbling on plastid protein biosynthesis, we analyzed plastid protein accumulation and several photosynthetic parameters that serve as proxy for chloroplast translational activity [10], [20], [21], [30]. Consistent with the severity of the mutant phenotypes (Figure 5), accumulation of the plastid-encoded large subunit of ribulose-1,5-bisphosphate carboxylase (Rubisco), by far the most abundant protein in green tissues (accumulating to more than 50% of the plant's total soluble protein), was most strongly reduced in the Δ*trnT-GGU* plants and, to a lesser extent, also in the Δ*trnS-GGA* plants, whereas the Δ*trnL-CAA* and Δ*trnV-GAC* knock-out mutants were much less affected (Figure 6A). Analysis of electrophoretically separated total soluble protein by Coomassie staining confirmed the underrepresentation of the (usually highly abundant) plastid-encoded proteins in the Δ*trnT-GGU* and Δ*trnS-GGA* plants, as evidenced by the high intensity background staining, which largely comes from lowly abundant cytosolic proteins (Figure 6A).

As the vast majority of plastid-encoded gene products is either directly or indirectly involved in photosynthesis, impaired plastid gene expression is usually well correlated with reduced photosynthetic activity [10], [20], [21], [30]. We, therefore, measured chlorophyll content, chlorophyll a:b ratio and the maximum quantum efficiency of photosystem II (F_V_/F_M_) in the homoplasmic tRNA knock-out mutants. As expected, these analyses revealed a good correlation between the severity of the mutant phenotype (Figure 5) and the degree of impairment in these key photosynthetic parameters. When young developing leaves were examined (where the demand for chloroplast protein biosynthesis is highest due to the costly *de novo* synthesis of the thylakoidal protein complexes; [20], [21]), the Δ*trnT-GGU* plants were most strongly affected in all three parameters, followed by the Δ*trnS-GGA* plants. This was also the case in mature plants, with the notable exception that the chlorophyll content was higher in the Δ*trnT-GGU* plants than in the Δ*trnS-GGA* plants (Figure 6B). Taken together, these results confirm that the synthesis rate of photosynthetic protein complexes is significantly reduced in the chloroplasts of two mutants exhibiting pronounced mutant phenotypes. Our data also suggest that the effects of enforced superwobbling can be variable during development in a codon box-dependent manner.

To confirm that superwobbling results in incorporation of the correct amino acid into the polypeptide chain, we determined peptide sequences from the plastid genome-encoded large subunit of Rubisco (RbcL) in the mutant with the strongest phenotype, the Δ*trnT-GGU* plants, using mass spectrometric methods [10]. The detected peptides covered 19 out of 23 threonine triplets in RbcL, whose decoding is dependent on superwobbling in the Δ*trnT-GGU* mutant (Figure S9). For 17 residues, correct incorporation of threonine was additionally verified by *de novo* amino acid sequencing. At all positions, only threonine was found to be incorporated into the Rubisco protein, suggesting a high accuracy of codon reading by superwobbling.

## Discussion

**Figure 7:**
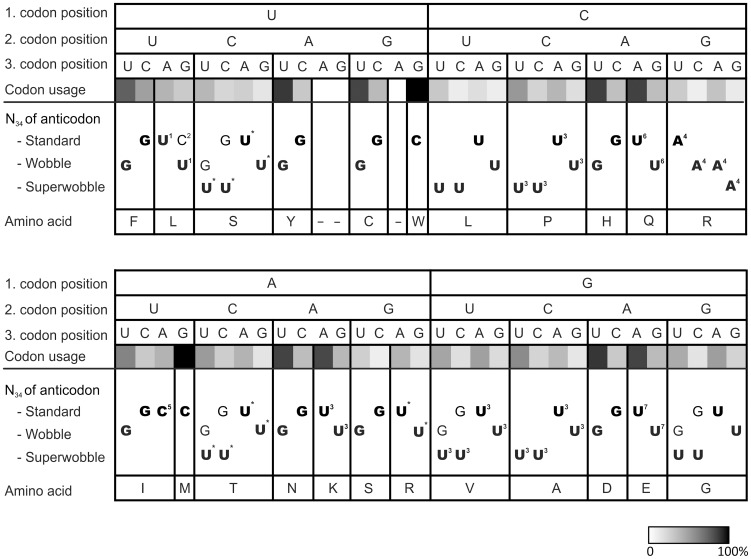
Decoding of the 64 triplets of the genetic code in plastids. Codon recognition by standard Watson-Crick base pairing, wobbling and/or superwobbling is indicated by the nucleotide in the wobble position of the anticodon of the tRNA species that can decode the triplet. Essential tRNA species are indicated in bold, non-essential tRNAs in normal font. The codon usage in plastids of *Nicotiana tabacum* is shown on a greyscale. Superscript numbers and indices indicate nucleoside modifications in the wobble position (N_34_) of the anticodon of the tRNA species. ^1^: 2′-O-methyluridine [26]; ^2^: 2′-O-methylcytidine [26]; ^3^: unknown modification [23], [45], [46], [47]; ^4^: inosine [28]; ^5^: lysidine [48], [49]; ^6^: 5-carboxymethylaminomethyl uridine (cmnm^5^U; [50]); ^7^: 5-methylaminomethyl-2-thiouridine (mam^5^s^2^U; [51]; http://trnadb.bioinf.uni-leipzig.de/); ^*^: modification status of the wobble uridine unknown (RNA sequence not determined); -: stop codon.

In the course of this work, we have generated a set of wobbling and superwobbling mutants that allowed us to derive the complete set of decoding rules for a prokaryotic system, the chloroplast of higher plants (Figure 7). The genetic system of higher plant chloroplasts has the important advantage over bacterial systems that reduced translational activity results in readily visible phenotypes that are easily quantifiable (Figure 5 and Figure 6). We have shown here that (i) superwobbling is not restricted to strong codons (in the sense of the Lagerkvist hypothesis; [15], [16]), (ii) superwobbling in mixed codons can be even more efficient than superwobbling in strong codons, and (iii) the efficiency of superwobbling varies greatly in a codon box-dependent manner (and apparently does not correlate with the number of hydrogen bonds established between codon and anticodon).

The functional and evolutionary significance of tRNA gene reduction facilitated by superwobbling is exemplified by the decoding in the threonine (ACN) box (Figure 1 and Figure 5). The *trnT-GGU* gene shown to be non-essential in this study is missing from the plastid genomes of presumably all species in the family Geraniaceae [31]. Our data suggest that the loss of this gene can be largely compensated by superwobbling of the second threonine isoacceptor, the tRNA-Thr(UGU). However, although our results provide a mechanistic framework for the dispensability of tRNA-Thr(GGU), the reduced fitness of the Δ*trnT-GGU* plants indicates that additional genetic adaptations were likely required in Geraniaceae evolution to make the loss of the *trnT-GGU* gene neutral. Whether these additional changes lie in the codon usage, the structure of the chloroplast ribosome or the structure and/or modification pattern of the tRNA-Thr(UGU), remains to be determined. It is noteworthy in this respect that, in addition to the anticodon itself, sequence features in the anticodon loop and stem (the so-called “extended anticodon”) have been suggested to contribute to the efficiency of decoding [32].

We have also investigated the curious case of the two leucine codons UUA and UUG, which represent the only codon pair that is read by two distinct tRNA species, tRNA-Leu(CAA) and tRNA-Leu(UAA). Thus, this appeared to be the only case of avoided wobbling and preferred decoding by standard Watson-Crick base pairing. However, non-essentiality of the *trnL-CAA* gene and lack of a discernible phenotype of the Δ*trnL-CAA* plants suggests that there is neither a requirement nor an obvious selective advantage of decoding UUA and UUG triplets by standard base pairing. This finding raises the question why a gene for tRNA-Leu(CAA) was retained in the plastid genome. We propose here that this is due to the location of the *trnL-CAA* gene in the inverted repeat region of the plastid genome. Due to copy correction by gene conversion [33], [34], the inverted repeat region is known to have a much lower mutation rate than the two single-copy regions of the plastid genome. This effect makes gene losses by mutational degeneration less probable and may be responsible for the retention of the *trnL-CAA* gene. It could also explain retention of the *trnV-GAC* gene, despite its seeming expendability due to efficient superwobbling by the tRNA-Val(UAC) isoacceptor (Figure 5 and Figure 6). The *trnV-GAC* gene is also located in the inverted repeat region of the genome and, thus, copy correction by gene conversion may have prevented its evolutionary loss. Alternatively, these seemingly dispensable tRNA species could be maintained because they confer a selective advantage under specific environmental conditions, such as abiotic stress conditions known to impact plastid translation [20].

Our results reported here show that superwobbling can participate in the decoding of all four-fold degenerate codon boxes (Figure 7). Although the phenotypes of some of our tRNA knock-out mutants indicate that superwobbling is, at least in some codon boxes, less efficient than standard and wobble base pairing, our data suggest that superwobbling is also used in the presence of the tRNA isoacceptor that reads the codons with a pyrimidine in third position by Watson-Crick base pairing and wobbling. Thus, the tRNA isoacceptor with uridine in the wobble position significantly contributes to the reading of triplets with pyrimidines in third codon position *in vivo*. Based on these findings and previous analyses of essential plastid tRNA genes [35], [10], [22], we have compiled a revised table of the decoding rules in plastids (Figure 7). It shows the codon reading by Watson-Crick base pairing, wobbling and superwobbling, which is now supported by a complete set of experimental data for all codon boxes, in which superwobbling is theoretically possible. Essentiality of both tRNA-Ile species (encoded by the plastid *trnI-GAU* and *trnI-CAU* genes) for reading of the three isoleucine codons (AUU, AUC and AUA) was demonstrated previously [22]. Figure 7 also includes information on tRNA essentiality and relates the decoding rules to the codon usage in the plastid genome [36]. The latter analysis shows that the codon usage is much stronger correlated with the (generally high) AT content in the plastid DNA than with the mode of triplet reading. For example, in the three codon boxes read by a single tRNA species with a uridine in the wobble position of the anticodon (the CCN proline box, the CUN leucine box and the GCN alanine box), the triplets with U and A in third codon position are most frequently used, even though the triplet with U in third position can only be read by superwobbling.

In all four-fold degenerate codon boxes, the tRNA species with uridine in the wobble position turned out to be essential (Figure 7; Figures S1, S2, S3, S4, S5, S6, S7, S8), indicating that two-out-of-three reading [15], [16] by the isoacceptor with guanosine in the wobble position is not possible. This is in agreement with previous reasoning that, for steric reasons, G-G and G-A base pairing in the wobble position is unlikely to occur [1]. Finally, our experimental data suggest that the minimum set of tRNAs comprises 25 tRNA species (Figure 7; additionally including the initiator tRNA-fMet), rather than 26 as proposed recently [9]. This minimum number holds under the assumptions that the standard genetic code is used and no unorthodox tRNA modifications occur, while maximum use is made of wobbling and superwobbling.

## Materials and Methods

### Plant material, growth conditions, and phenotypic analyses

To generate leaf material for chloroplast transformation experiments, tobacco (*Nicotiana tabacum* cv. Petit Havana) plants were raised under aseptic conditions on agar-solidified Murashige and Skoog medium (MS medium) containing 30 g/L sucrose [37]. Transplastomic lines were rooted and propagated on MS medium supplemented with 500 mg/L spectinomycin. For seed production and analysis of plant phenotypes, transplastomic plants were grown in soil under standard greenhouse conditions (relative humidity: 55%; day temperature: 25°C; night temperature: 20°C; diurnal cycle: 16 h light and 8 h darkness; light intensity ∼200 µE m^−2^ s^−1^). For growth assays under low-light conditions, light intensities of ∼80 µE m^−2^ s^−1^ were used. Inheritance patterns and seedling phenotypes were analyzed by germination of surface-sterilized seeds on MS medium with 500 mg/L spectinomycin.

### Construction of plastid transformation vectors

Vectors for the targeted knock-out of tRNA genes were constructed by inserting or replacing the respective genes with an *aadA* cassette in cloned plastid DNA fragments. For knock-out of the *trnA*, *trnR* and *trnT* genes, a cassette comprising the *aadA* coding region from *Escherichia coli*, the *Prrn* promoter and the *psbA* 3′ UTR from *Nicotiana tabacum* [17] was used. For inactivation of the *trnL*, *trnS* and *trnV* genes, an *aadA* cassette derived from plasmid pLS1 (comprising the *aadA* coding region from *E. coli*, the *psbA* promoter and the *rbcL* 3′ UTR from *Chlamydomonas reinhardtii*; [21]) was used. All plastid transformation vectors constructed for this study were verified by restriction analysis and DNA sequencing. For construction of vector pΔ*trnA-UCG*, the genomic region surrounding the *trnA-UCG* gene (corresponding to nucleotide positions 135,448 to 138,542 of the *N. tabacum* plastid genome; GenBank accession number NC_001879; [38]) was excised from a cloned SalI restriction fragment with HincII and EcoRI and inserted into the cloning vector pUC18 digested with the same enzymes. The resulting plasmid clone was digested with Ecl136II and AgeI, treated with T4 DNA polymerase to produce blunt ends and ligated to the *aadA* cassette [17] excised from a plasmid clone with Ecl136II and DraI. For construction of vector pΔ*trnL-CAA*, the genomic region surrounding the *trnL-CAA* gene (corresponding to nucleotide positions 95,356 to 98,153 of the *N. tabacum* plastid genome) was subcloned as PstI/SalI fragment and inserted into the cloning vector pBS KS+ digested with the same enzyme combination. The resulting plasmid clone was digested with EcoNI and EcoRV, treated with T4 DNA polymerase to generate blunt ends and ligated to the *aadA* cassette excised from pLS1 [21] with SmaI. For construction of vector pΔ*trnL-UAG*, a fragment from the *N. tabacum* plastid genome (corresponding to nucleotide positions 115,142 to 117,087) was amplified with the primers P5_trnLUAGFRAGFOR and P3_trnLUAGFRAGREV (for primer sequences, see Table S2). The PCR product was inserted into the cloning vector pUC18 digested with the HincII. The resulting plasmid clone was digested with AccI (to linearize it within the *trnL-UAG* gene), treated with T4 DNA polymerase to generate blunt ends and ligated to the *aadA* cassette excised from pLS1 with SmaI. Vector pΔ*trnL-UAA* was constructed by amplifying the *trnL-UAA* containing region (corresponding to nucleotide positions 48,624 to 50,543) with primers P5_trnLUAAmod1 and P3_trnLUAAmod2. The obtained PCR fragment was cloned into pUC18 digested with HincII. To replace the *trnL-UAA* gene with the *aadA* cassette, a PCR strategy was used. An initial PCR amplification was done with primers P5_trnLUAAmod1 and P3_trnLUAAmod3 and the pUC18/HincII clone as template to obtain the left flank of the *trnL-UAA* region. Another PCR was performed with primers P5_trnLUAAmod4 and P3_trnLUAAmod2 using the same plasmid as template to amplify the right flank of the *trnL-UAA* region. A third PCR amplified the *aadA* cassette (with additional sequences complementary to the flanking plastome sequences of *trnL-UAA* introduced with the primer sequences) using the primer pair P5_trnLUAAaadAkass5 and P3_trnLUAAaadAkass6 and pLS1 as template. In a final PCR, the three PCR products were combined and used as templates for amplification with primers P5_trnLUAAmod1 and P3_trnLUAAmod2, resulting in a knock-out allele in which *trnL-UAA* is substituted by the *aadA* cassette. The amplification product was cloned into pUC18 digested with HincII. For construction of vector pΔ*trnR-UCU*, the respective genomic region (corresponding to nucleotide positions 7,924 to 10,206 of the *N. tabacum* plastid genome) was subcloned as StuI/SalI fragment and inserted into the cloning vector pBS KS+ digested with EcoRV and SalI. The resulting plasmid clone was digested with KpnI and SalI and ligated to the genomic region surrounding the *trnR-UCU* gene (corresponding to nucleotide positions 10,207 to 12,066 and excised from a library clone with the same two enzymes). The resulting vector was linearized with PshAI and ligated to ligated to the *aadA* cassette [17] excised from a plasmid clone with Ecl136II and DraI. For construction of the vector pΔ*trnR-ACG*, a fragment from the *N. tabacum* plastid genome (corresponding to nucleotide positions 131,485 to 133,119) was subcloned with ApaI and StuI and inserted into the cloning vector pBS KS+ digested with ApaI and EcoRV. This resulting plasmid clone was digested with PmlI to linearize it within the *trnR-ACG* gene, followed by insertion of the *aadA* cassette [17] as Ecl136II/DraI restriction fragment. For construction of the vector pΔ*trnS-UGA*, a plasmid clone containing the *trnG-GCC* and *trnS-UGA* genes (corresponding to nucleotide positions 36,834 to 38,889; [10]) was cut with BstBI, treated with T4 DNA polymerase to produce blunt ends and ligated to the *aadA* cassette excised from pLS1 with SmaI, resulting in disruption of the *trnS-UGA* gene. Vector pΔ*trnS-GGA* was constructed as follows: the region upstream of *trnS-GGA* (corresponding to nucleotide positions 46,432 to 47,125) was amplified with primers P5_ 5′ClaItrnS and P3_ 3′trnS (carrying a 5′ overhang complementary to the *aadA* cassette) using tobacco genomic DNA as template. A second PCR amplified the *aadA* cassette using the primers P5_5′aadAtrnS and P3_3′aadAtrnS (with overhangs that are complementary to the plastid DNA sequences flanking *trnS-GGA*) and pLS1 as template. A third PCR amplified the region downstream of *trnS-GGA* (corresponding to nucleotides 47,205 to 47,498) with primers P5_5′trnS and P3_3′Bsu36ItrnS and tobacco genomic DNA as template. In a final PCR, the three generated PCR products were combined and used as templates for amplification with primers P5_5′ClaItrnS and P3_3′Bsu36ItrnS to produce a knock-out allele of *trnS-GGA*, in which the tRNA gene was replaced by the *aadA* cassette. The resulting amplification product was cloned into the pCR*®*2.1*-*TOPO vector (Invitrogen, Darmstadt, Germany) via TA cloning. For construction of vector pΔ*trnS-GCU*, a plasmid containing the sequences flanking *trnG-UCC* and *trnS-GCU* (corresponding to nucleotide positions 7,839 to 9,994; [10]) was cut with PsrI, then treated with T4 DNA polymerase to generate blunt ends and ligated to the *aadA* cassette excised from pLS1 with SmaI to disrupt the *trnS-GCU* gene. For construction of vector pΔ*trnT-UGU*, the genomic region surrounding the *trnT-UGU* gene (corresponding to nucleotide positions 46,536 to 49,222) was subcloned as XhoI/EcoRI fragment and inserted into the similarly digested cloning vector pBS KS+. The resulting plasmid was linearized with BlpI, treated with T4 DNA polymerase to produce blunt ends and ligated to the *aadA* cassette excised as Ecl136II/DraI fragment [17]. Vector pΔ*trnT-GGU* was constructed by subcloning the genomic region surrounding the *trnT-GGU* gene (corresponding to nucleotide positions 32,277to 33,823) as EcoRI/XbaI fragment into the similarly cut cloning vector pBS KS+. The resulting plasmid was linearized with NcoI, the overhanging ends were filled in with T4 DNA polymerase and ligated to the *aadA* cassette (as Ecl136II/DraI fragment; [17]). Vector pΔ*trnV-GAC* was constructed by amplifying the genomic region containing *trnV-GAC* (corresponding to nucleotide positions 101,426 to 103,540) with the primer pair P5_trnvfor and P3_trnvrev using tobacco total DNA as template. The resulting PCR fragment was cloned into the SmaI site of pUC18. To replace *trnV-GAC* with *aadA*, the *aadA* cassette was amplified from pLS1 plasmid DNA with primers P5_trnV-GAC-aadA-hin and P3_trnV-GAC-aadA-rev (carrying overhangs complementary to the plastome sequences surrounding *trnV-GAC*). In a second PCR, the region upstream of *trnV-GAC* was amplified with primers P5_trnV-GAC-MfeI-hin and P3_trnV-GAC-MfeI-rev using genomic DNA as template. A third PCR amplified the region downstream of *trnV-GAC* with primers P5_trnV-GAC-BbvCI-hin and P3_trnV-GAC-BbvCI-rev. The resulting amplification products were used as templates for a final PCR using primers P5_trnV-GAC-MfeI-hin and P3_trnV-GAC-BbvCI-rev. The PCR product was digested with MfeI and BbvCI and inserted into the similarly cut pUC18 clone of the *trnV-GAC* genomic region. For construction of vector pΔ*trnV-UAC*, a fragment from the *N. tabacum* plastid genome (corresponding to nucleotide positions 53,421 to 55,429) was amplified with primers P5_trnV-UAC-for and P3_trnV-UAC-rev and cloned into vector pUC18 digested with HincII. The resulting plasmid was digested with AccI (to linearize it within the *trnV-UAC* gene), treated with T4 DNA polymerase to produce blunt ends and ligated to the *aadA* cassette excised from pLS1 with SmaI.

### Plastid transformation and selection of transplastomic lines

For plastid transformation, young leaves from aseptically grown tobacco plants were bombarded with plasmid-coated 0.6-µm gold particles using a helium-driven biolistic gun (PDS1000He; BioRad). Primary spectinomycin-resistant lines were selected on plant regeneration medium containing 500 mg/L spectinomycin [17], [39]. Spontaneous spectinomycin-resistant plants were eliminated by double selection tests on medium containing both spectinomycin and streptomycin (500 mg/L each; [17],[40]). Several independent transplastomic lines were produced for each construct and subsequently subjected to three to four additional rounds of plant regeneration on spectinomycin-containing medium to enrich the transformed plastid genome and select for homoplasmy [20].

### Isolation of nucleic acids and hybridization procedures

Total plant DNA was extracted from plants grown under spectinomycin selection *in vitro* by a cetyltrimethylammoniumbromide-based method [41]. For RFLP analysis, DNA samples were treated with restriction enzymes, separated in 0.8–1% agarose gels, and blotted onto Hybond N nylon membranes (GE Healthcare, Buckinghamshire, UK). Total plant RNA for northern blot experiments was extracted using the peqGOLD TriFast reagent (Peqlab) following the manufacturer's protocol. RNA samples were denatured, separated in denaturing formaldehyde-containing agarose gels (2%) and blotted onto Hybond N nylon membranes (GE Healthcare). For hybridization, [α^32^P]dCTP-labeled probes were produced by random priming (Multiprime DNA labeling kit; GE Healthcare). Prior to labeling, DNA fragments were purified by agarose gel electrophoresis followed by extraction from excised gel slices using the NucleoSpin Extract II kit (Macherey-Nagel). Hybridizations were performed at 65°C using standard protocols. The probes for RFLP analysis of putative transplastomic lines were prepared from restriction fragments or amplified PCR products. For analysis of the Δ*trnL-UAG* lines, the probe (corresponding to the nucleotides 116.280 to 117.079 in the plastid genome of *N. tabacum*) was excised from plasmid pΔ*trnL-UAG* with BamHI and EcoRI. For the Δ*trnL-UAA* lines, the probe (corresponding to positions 49.891 to 50.503) was excised from pΔ*trnL-UAA* using PstI and XcmI. For RFLP analysis of Δ*trnL-CAA* lines, the plastid genome sequence from positions 95,356 to 95,947 was excised from plasmid pΔ*trnL-CAA* with PstI and EcoRI and used as probe. A probe for analysis of Δ*trnS-UGA* lines (covering the genomic region from nucleotide positions 34,311 to 34,769) was prepared by PCR amplification with primers P5_sense and P3_antisense (Table S2). To produce a probe for analysis of the Δ*trnS-GCU* lines, the sequence corresponding to nucleotide positions 8,725 to 9,750 was excised from plasmid pΔ*trnS-GCU* with MfeI. For RFLP analysis of the Δ*trnS-GGA* lines, the probe corresponded to nucleotide positions 47,205 to 47,498 in the tobacco plastid genome and was prepared by PCR amplification with primers P5_5′trnS and P3_Bsu36ItrnS. The probe for analysis of Δ*trnV-GAC* lines (covering the genomic region from nucleotide positions 10,2769 to 10,4234) was generated by PCR amplification with primers P5_16SkomplettF and P3_16SkomplettR (Table S2). For the Δ*trnV-UAC* lines, the probe (corresponding to positions 54,811 to 55,247 in the plastid genome) was prepared by PCR amplification with primers P5_atpEprfor and P3_atpEprrev. For analysis of the Δ*trnT-UGU* lines, the probe corresponded to nucleotide positions 48,583 to 49,222 in the plastid genome and was excised from plasmid pΔ*trnT-UGU* with BlpI and EcoRI. A probe for analysis of the Δ*trnT-GGU* lines (covering the region from nucleotide positions 32,277 to 33,035) was excised from plasmid pΔ*trnT-GGU* with AvrII and EcoRI. The probes for northern blot analysis of *trnL-CAA*, *trnT-GGU* and *trnV-GAC* were generated by PCR amplification using specific oligonucleotides (P_trnL-CAA-for/P_trnL-CAA-rev, P_trnT-GGU-A/P_trnT-GGU-B and P_trnV-GAC-for/P_trnV-GAC-rev, respectively; Table S2). For strand-specific detection of *trnS-GGA* in northern blot analyses, a 5′ end-labeled oligonucleotide was used (labeled with [γ-^32^P]ATP and T4 polynucleotide kinase). A standard labeling reaction contained the oligonucleotide (50 ng), 1× reaction buffer (70 mM Tris HCl, 10 mM MgCl_2_, 5 mM DTT), 10 µCi [γ-^32^P]ATP and 5 U of T4 polynucleotide kinase. The reaction was carried out at 37°C for 30 min, followed by inactivation of the enzyme at 95°C for 5 min.

### Chloroplast isolation and RNA extraction from purified chloroplasts

30–40 g tobacco leaves (harvested from 4–5 week old plants) were homogenized in 300 ml Grinding Buffer (GB; 0.4 M sorbitol, 50 mM HEPES, 2 mM EDTA, 0.1% BSA, 0.1% isoascorbat, pH 8.0) in a Waring Blender. The homogenate was filtrated through four layers of absorbent gauze and one layer of Miracloth (Calbiochem, San Diego, CA). The filtrate was centrifuged at 2,500×g for 3 min and the chloroplast pellet was dissolved in 1–2 ml GB, loaded onto Percoll gradients (40%–80% v/v in GB; Percoll™; GE Healthcare) and centrifuged at 13,300×g for 14 min. The intact chloroplasts were collected, washed with GB and pelleted by centrifugation at 2,000×g for 2 min. RNA was extracted with peqGOLD TriFast (Peqlab) following the manufacturer's instructions.

### Protein extraction, Western blot analysis, and mass spectrometric analysis

Total soluble protein was extracted from leaf samples using published procedures [42]. The protein concentration of the extracts was determined using the Bradford assay (Roth, Karlsruhe, Germany) and known concentrations of bovine serum albumin as standard. Protein samples were separated by electrophoresis in 15% SDS-containing polyacrylamide gels, and the proteins in the gel were directly visualized by Coomassie blue staining. For immunoblot analysis, protein gels were blotted onto polyvinylidene fluoride (PVDF) membranes (Hybond-P; GE Healthcare). For detection of the large subunit of Rubisco, a polyclonal antibody (raised in rabbits; Agrisera, Vännäs, Sweden) was used. The chemiluminescence detection reaction was carried out with the ECL Plus western blotting detection system (GE Healthcare). Mass spectrometric determination of Rubisco peptide sequences was performed as described previously [10].

### Physiological measurements

Chlorophyll contents were determined in 80% (v/v) acetone using published methods [43]. PSII quantum efficiency (F_v_/F_m_) was determined using a pulse-amplitude modulated fluorimeter (DUAL-PAM-100; Heinz Walz GmbH). Prior to the measurments, plants were dark adapted for 20 min.

## Supporting Information

Figure S1 Targeted disruption of the plastid *trnA-UGC* gene. (A) Physical map of the *trnA-UGC*-containing region in the tobacco plastid genome (ptDNA). (B) Map of the transformed plastid genome (transplastome) produced with plastid transformation vector pΔ*trnA-UGC*. The *aadA* cassette is shown as grey box. (C) Map of the recombination product in Δ*trnA-UGC-Rc* transplastomic lines. As shown in earlier studies, recombination occurs between the 3′ UTR of the *aadA* cassette and that of the endogenous *psbA* gene [19], [20]. Genes above the line are transcribed from the left to the right, genes below the line are transcribed in the opposite direction. Selected restriction sites used for cloning and RFLP analysis are indicated. The hybridization probe and the expected sizes of detected DNA fragments are also shown. Introns are represented by open boxes. (D) RFLP analysis of Δ*trnA-UGC* plastid transformants. The transplastomic lines remain heteroplasmic and show both the 3.2 kb wild type-specific hybridization band and the 6 kb band resulting from flip-flop recombination between the 3′ UTR of the *aadA* and that of the endogenous *psbA* gene [19]. Wt: wild type. (E) Seed assay confirming heteroplasmy of the Δ*trnA-UGC* plants. The transplastome is lost from most seedlings as evidenced by their white phenotype upon germination on spectinomycin-containing medium. The arrow points to a green (spectinomycin-resistant) seedling that still harbors the transplastome. (F) Phenotype of typical heteroplasmic Δ*trnA-UGC* plants. Three Δ*trnA-UGC* plants (right) and a wild-type plant (left) after transfer to soil and continued growth under greenhouse conditions are shown. Misshapen leaves with missing sectors indicate essentiality of the *trnA-UGC*.
(TIF)

Figure S2 Targeted disruption of the plastid *trnS-UGA* gene. (A) Physical map of the region in the tobacco plastid DNA containing the *trnS-UGA* gene. Genes above the line are transcribed from the left to the right, genes below the line are transcribed in the opposite direction. The bent arrows indicate the borders of the transformation plasmid. Restriction sites used for RFLP analysis are indicated. The hybridization probe and the expected size of the detected restriction fragment are also shown. (B) Map of the transplastome produced with plastid transformation vector pΔ*trnS-UGA*. The *aadA* marker gene is shown as grey box. (C) RFLP analysis of Δ*trnS-UGA* transplastomic plants. The transplastomic lines remain heteroplasmic and show both the wild type-specific 5 kb restriction fragment and the 6.6 kb band diagnostic of the transplastome. Wt: wild type. (D) Leaf phenotype of a typical heteroplasmic Δ*trnS-UGA* plant. Arrows point to misshapen leaves. (E) Segregation analysis of a Δ*trnS-UGA* plant. Seeds from a selfed transplastomic plant were sown on spectinomycin-containing synthetic medium. Spectinomycin sensitivity of most seedlings suggests a strong tendency to lose the transgenic plastid genome in the absence of antibiotic selection. A single spectinomycin-resistant seedling (that has retained the transplastome) is indicated by the arrow.
(TIF)

Figure S3 Targeted inactivation of the plastid *trnS-GCU* gene. (A) Physical map of the region in the tobacco plastid genome containing *trnS-GCU*. Genes above the line are transcribed from the left to the right, genes below the line are transcribed in the opposite direction. The bent arrows indicate the borders of the transformation vector. The restriction sites used for RFLP analysis are indicated. The hybridization probe and the expected size of detected DNA fragment are also shown. Introns are represented by open boxes. (B) Map of the transformed plastid genome obtained with plastid transformation vector pΔ*trnS-GCU*. The *aadA* selectable marker gene is shown in grey. (C) RFLP analysis of Δ*trnS-GCU* plastid transformants. The transplastomic lines remain heteroplasmic and show both the 3.9 kb hybridization band diagnostic of the wild-type plastid genome and the 5.5 kb band diagnostic of the transplastome. Wt: wild type. (D) Leaf-loss phenotype of a typical heteroplasmic Δ*trnS-GCU* plant. A misshapen leave is indicated by the arrow. (E) Inheritance assay of a Δ*trnS-GCU* plant. An example of a spectinomycin-resistance seedling that has retained copies of the transplastome is indicated by the arrow.
(TIF)

Figure S4 Targeted inactivation of the plastid *trnV-UAC* gene. (A) Physical map of the region in the tobacco plastid genome containing *trnV-UAC*. Genes above the line are transcribed from the left to the right, genes below the line are transcribed in the opposite direction. The bent arrows indicate the borders of the transformation plasmid. The restriction sites used for RFLP analysis are indicated. The hybridization probe and the expected size of the detected DNA fragment are also shown. The introns in *trnV-UAC* is represented by an open box. (B) Map of the transformed plastid genome (transplastome) produced with plastid transformation vector pΔ*trnV-UAC*. The *aadA* marker is shown as grey box. (C) RFLP analysis of Δ*trnV-UAC* plastid transformants. The transplastomic lines remain heteroplasmic and show a stable ratio of the wild type-specific 1.8 kb band and the 3.3 kb band diagnostic of the transplastome. Wt: wild type. (D) Phenotype of a typical heteroplasmic Δ*trnV-UAC* plant. The arrow points to an example of a misshapen leaf that lacks part of the leaf blade. (E) Inheritance assay of a Δ*trnV-UAC* plant. Spectinomycin-resistance seedling that have retained copies of the transplastome are green, seedlings that have lost all copies of the transgenic plastid genome are white.
(TIF)

Figure S5 Targeted inactivation of the plastid *trnL-UAA* gene. (A) Physical map of the region in the tobacco plastid genome (ptDNA) containing the *trnL-UAA* gene. Genes above the line are transcribed from the left to the right, genes below the line are transcribed in the opposite direction. The bent arrows indicate the borders of the transformation plasmid. Restriction sites used for RFLP analysis are indicated. The hybridization probe and the expected size of the detected DNA fragment are also shown. The intron in the *trnL-UAA* gene is represented by an open box. (B) Map of the transformed plastid genome (transplastome) produced with plastid transformation vector pΔ*trnL-UAA*. The *aadA* cassette is shown as grey box. (C) RFLP analysis of Δ*trnL-UAA* plastid transformants. The transplastomic lines remain heteroplasmic and show a stable ratio of the 1.6 kb hybridizing fragment diagnostic of the wild-type ptDNA and the 3.1 kb band diagnostic of the transformed ptDNA. Wt: wild type. (D) Phenotype of a typical heteroplasmic Δ*trnL-UAA* plant. The arrow points to an example of a misshapen leaf. (E) Segregation analysis of a Δ*trnL-UAA* plant. Seeds from a selfed transplastomic plant were sown on spectinomycin-containing synthetic medium. Spectinomycin sensitivity of most seedlings indicates that the plants tend to rapidly lose the transplastome in the absence of antibiotic selection. An example of a green spectinomycin-resistant seedling (that has retained the transplastome) is indicated by the arrow.
(TIF)

Figure S6 Targeted disruption of the plastid *trnL-UAG* gene. (A) Physical map of the region in the tobacco plastid genome containing the *trnL-UAG* gene. Genes above the line are transcribed from the left to the right, genes below the line are transcribed in the opposite direction. The bent arrows indicate the borders of the transformation plasmid. Restriction sites used for RFLP analysis are indicated. The hybridization probe and the expected size of detected DNA fragment are also shown. (B) Map of the transformed plastid genome obtained with plastid transformation vector pΔ*trnL-UAG*. The *aadA* selectable marker cassette is shown as grey box. (C) RFLP analysis of Δ*trnL-UAG* chloroplast transformants. The transplastomic lines remain heteroplasmic and show both the wild type-specific 2.1 kb band and the transplastome-specific 3.6 kb band. Wt: wild type. (D) Phenotype of a typical heteroplasmic Δ*trnL-UAG* plant. The arrow points to an example of a misshapen leaf that lacks part of the leaf blade. (E) Example of a seed assay confirming heteroplasmy of Δ*trnL-UAG* plants and gradual loss of the transplastome in the absence of antibiotic selection. The transplastome is lost from most seedlings as evidenced by their white phenotype upon germination on spectinomycin-containing synthetic medium. The arrows points to two green (spectinomycin-resistant) seedlings that still harbor copies of the transplastome.
(TIF)

Figure S7 Targeted disruption of the plastid *trnR-ACG* gene. (A) Physical map of the region in the tobacco plastid genome containing the *trnR-ACG* gene. Genes above the line are transcribed from the left to the right, genes below the line are transcribed in the opposite direction. Selected restriction sites used for cloning and RFLP analysis are indicated. The hybridization probe and the expected sizes of detected DNA fragments are also shown. Introns are represented by open boxes. (B) Map of the transformed plastid genome (transplastome) produced with plastid transformation vector pΔ*trnR-ACG*. The *aadA* cassetten is shown as grey box. (C) Map of the recombination product (Δ*trnR-ACG-Rc*) between the 3′ UTR the *aadA* cassette and that of the endogenous *psbA* gene. (D) RFLP analysis of Δ*trnR-ACG* plastid transformants. All transplastomic lines remain heteroplasmic and show both the 3.5 kb wild type-specific hybridization band and a band diagnostic of the transplastome. The 7.3 kb band appearing in addition to or instead of the expected 4.7 kb transplastomic fragment in the transformants is the result of flip-flop recombination between the 3′ UTR of the *aadA* and that of the endogenous *psbA* gene [19]. Wt: wild type. (E) Inheritance assay of a Δ*trnR-ACG* plant. Spectinomycin-resistance seedlings that have retained the transplastome are green on antibiotic-containing medium. The phenotype of heteroplasmatic Δ*trnR-ACG* lines is shown in Figure S8E.
(TIF)

Figure S8 Targeted disruption of the plastid *trnR-UCU* gene. (A) Physical map of the region in the tobacco plastid genome containing the *trnR-UCU* gene. Genes above the line are transcribed from the left to the right, genes below the line are transcribed in the opposite direction. Selected restriction sites used for cloning and RFLP analysis are indicated. The hybridization probe and the expected size of detected DNA fragments are also shown. Introns are represented by open boxes. (B) Map of the transformed plastid genome (transplastome) produced with plastid transformation vector pΔ*trnR-UCU*. The *aadA* marker cassette is shown as grey box. (C) RFLP analysis of Δ*trnR-UCU* plastid transformants. The transplastomic lines remain heteroplasmic and show both the 5.4 kb wild type-specific hybridization band and the 6.6 kb band diagnostic of the transplastome. Wt: wild type. (D) Inheritance assay of a Δ*trnR-UCU* plant. Spectinomycin-resistance seedlings that have retained the transplastome are green on antibiotic-containing medium. (E) Phenotype of heteroplasmic Δ*trnR-UCU* and Δ*trnR-ACG* plants. A wild-type plant (left), two Δ*trnR-UCU* plants (middle) and two Δ*trnR-ACG* plants grown under greenhouse conditions are shown. Misshapen leaves with missing sectors indicate essentiality of the *trnR-UCU* and Δ*trnR-ACG* genes.
(TIF)

Figure S9 Verification of threonine incorporation into the large subunit of Rubisco (RbcL) in the Δ*trnT-GGU* plants by MS/MS. (A) Detected RbcL peptides and threonine residues dependent on superwobbling in the Δ*trnT-GGU* mutant. The RbcL peptides detected by mass spectrometry are marked in bold. The threonines encoded by ACU and ACC codons are indicated in blue and green, respectively. These codons are decoded by tRNA-Thr(GGU) in the wild type, but are read by tRNA-Thr(UGU) in the Δ*trnT-GGU* transplastomic lines using superwobbling. In all detected peptides, threonine was correctly incorporated. (B) Confirmation of threonine incorporation into RbcL by *de novo* sequencing. Example of a y-ion series from a peptide containing an ACC-encoded threonine from the wild-type sample. (C) Example of a y-ion series from a peptide containing an ACC-encoded threonine from a Δ*trnT-GGU* plant.
(TIF)

Table S1 Numbers of codons in the plastid genome affected by the knock-out of the four non-essential tRNA genes *trnL-CAA*, *trnS-GGA*, *trnT-GGU* and *trnV-GAC*. The total numbers of codons for leucine, serine, threonine and valine in all plastid protein-coding genes are given as well as the numbers of codons that are optimally decoded by each of the knocked-out tRNA species.
(DOC)

Table S2 Sequences of oligonucleotides used for cloning and/or generation of hybridization probes. Sequences complementary to sequences in the *aadA* cassette are underlined.
(DOC)
