# Supplementary figures and images for: The Contributions of Wobbling and Superwobbling to the Reading of the Genetic Code
Source: PLoS Genet. 2012 Nov 15;8(11):e1003076. doi: 10.1371/journal.pgen.1003076 (PMC3499367; doi:10.1371/journal.pgen.1003076)

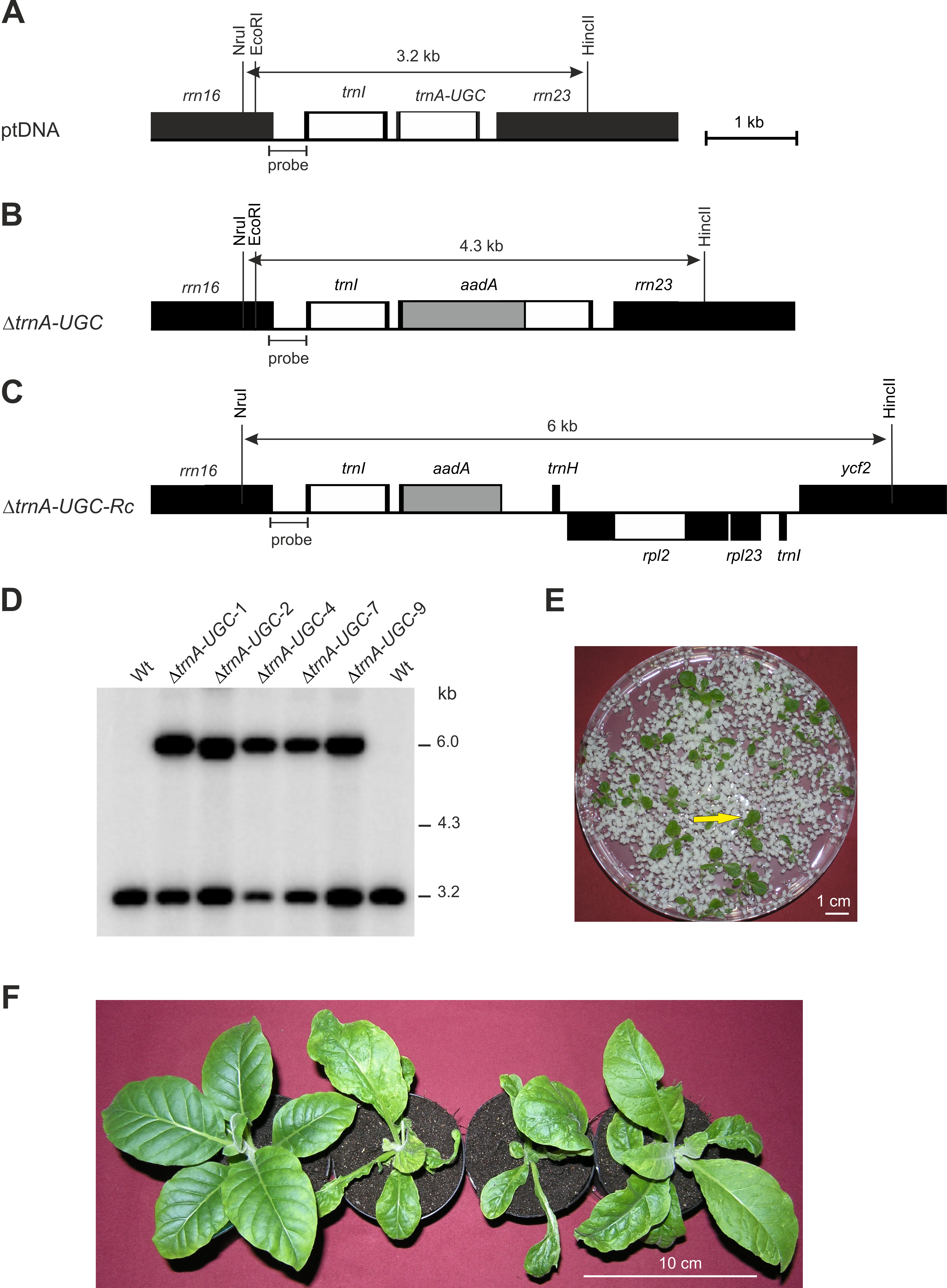

Supplement: Figure S1 — Targeted disruption of the plastid trnA-UGC gene. (A) Physical map of the trnA-UGC-containing region in the tobacco plastid genome (ptDNA). (B) Map of the transformed plastid genome (transplastome) produced with plastid transformation vector pΔtrnA-UGC. The aadA cassette is shown as grey box. (C) Map of the recombination product in ΔtrnA-UGC-Rc transplastomic lines. As shown in earlier studies, recombination occurs between the 3′ UTR of the aadA cassette and that of the endogenous psbA gene [19], [20]. Genes above the line are transcribed from the left to the right, genes below the line are transcribed in the opposite direction. Selected restriction sites used for cloning and RFLP analysis are indicated. The hybridization probe and the expected sizes of detected DNA fragments are also shown. Introns are represented by open boxes. (D) RFLP analysis of ΔtrnA-UGC plastid transformants. The transplastomic lines remain heteroplasmic and show both the 3.2 kb wild type-specific hybridization band and the 6 kb band resulting from flip-flop recombination between the 3′ UTR of the aadA and that of the endogenous psbA gene [19]. Wt: wild type. (E) Seed assay confirming heteroplasmy of the ΔtrnA-UGC plants. The transplastome is lost from most seedlings as evidenced by their white phenotype upon germination on spectinomycin-containing medium. The arrow points to a green (spectinomycin-resistant) seedling that still harbors the transplastome. (F) Phenotype of typical heteroplasmic ΔtrnA-UGC plants. Three ΔtrnA-UGC plants (right) and a wild-type plant (left) after transfer to soil and continued growth under greenhouse conditions are shown. Misshapen leaves with missing sectors indicate essentiality of the trnA-UGC. (TIF) [file pgen.1003076.s001.tif]

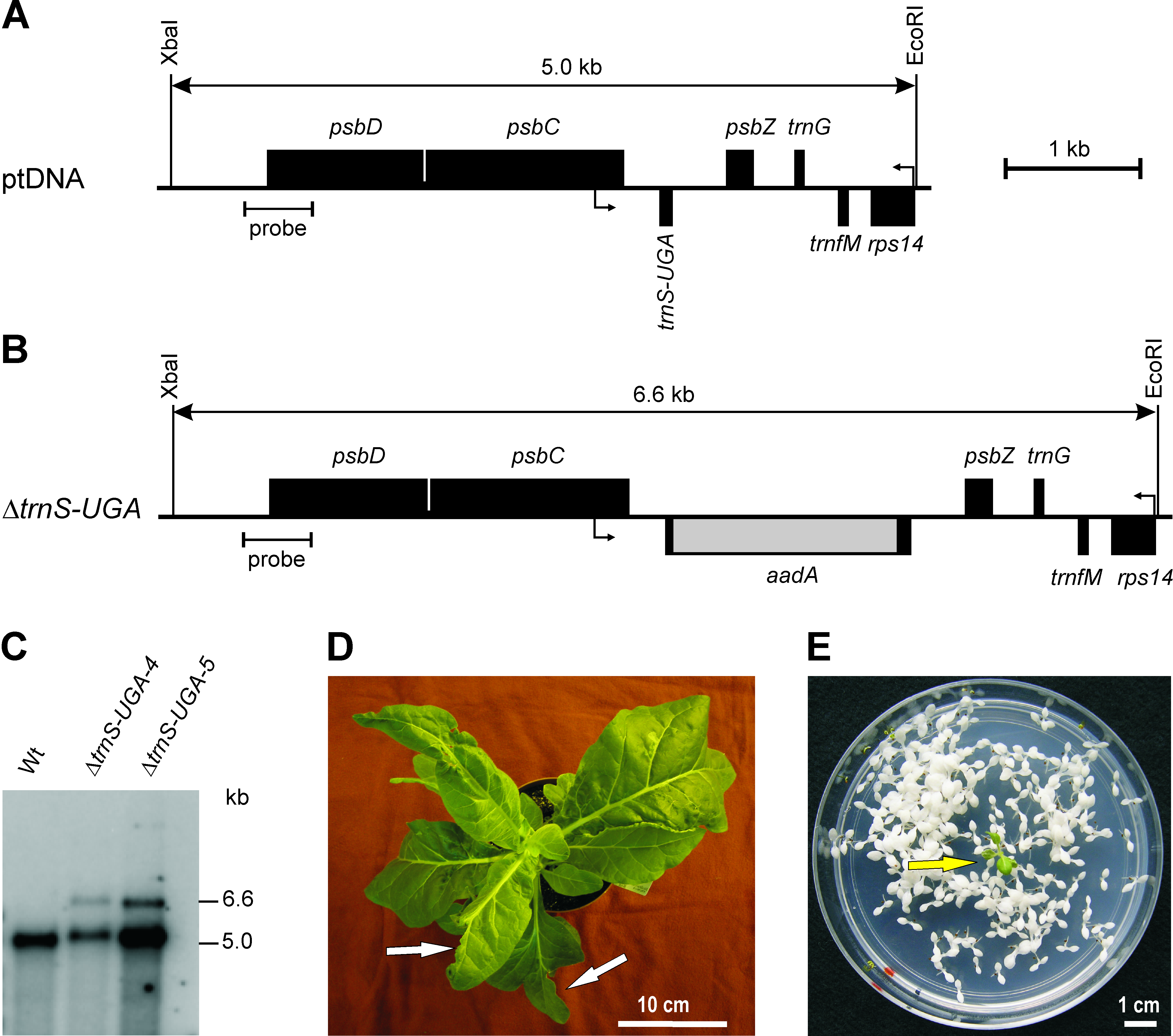

Supplement: Figure S2 — Targeted disruption of the plastid trnS-UGA gene. (A) Physical map of the region in the tobacco plastid DNA containing the trnS-UGA gene. Genes above the line are transcribed from the left to the right, genes below the line are transcribed in the opposite direction. The bent arrows indicate the borders of the transformation plasmid. Restriction sites used for RFLP analysis are indicated. The hybridization probe and the expected size of the detected restriction fragment are also shown. (B) Map of the transplastome produced with plastid transformation vector pΔtrnS-UGA. The aadA marker gene is shown as grey box. (C) RFLP analysis of ΔtrnS-UGA transplastomic plants. The transplastomic lines remain heteroplasmic and show both the wild type-specific 5 kb restriction fragment and the 6.6 kb band diagnostic of the transplastome. Wt: wild type. (D) Leaf phenotype of a typical heteroplasmic ΔtrnS-UGA plant. Arrows point to misshapen leaves. (E) Segregation analysis of a ΔtrnS-UGA plant. Seeds from a selfed transplastomic plant were sown on spectinomycin-containing synthetic medium. Spectinomycin sensitivity of most seedlings suggests a strong tendency to lose the transgenic plastid genome in the absence of antibiotic selection. A single spectinomycin-resistant seedling (that has retained the transplastome) is indicated by the arrow. (TIF) [file pgen.1003076.s002.tif]

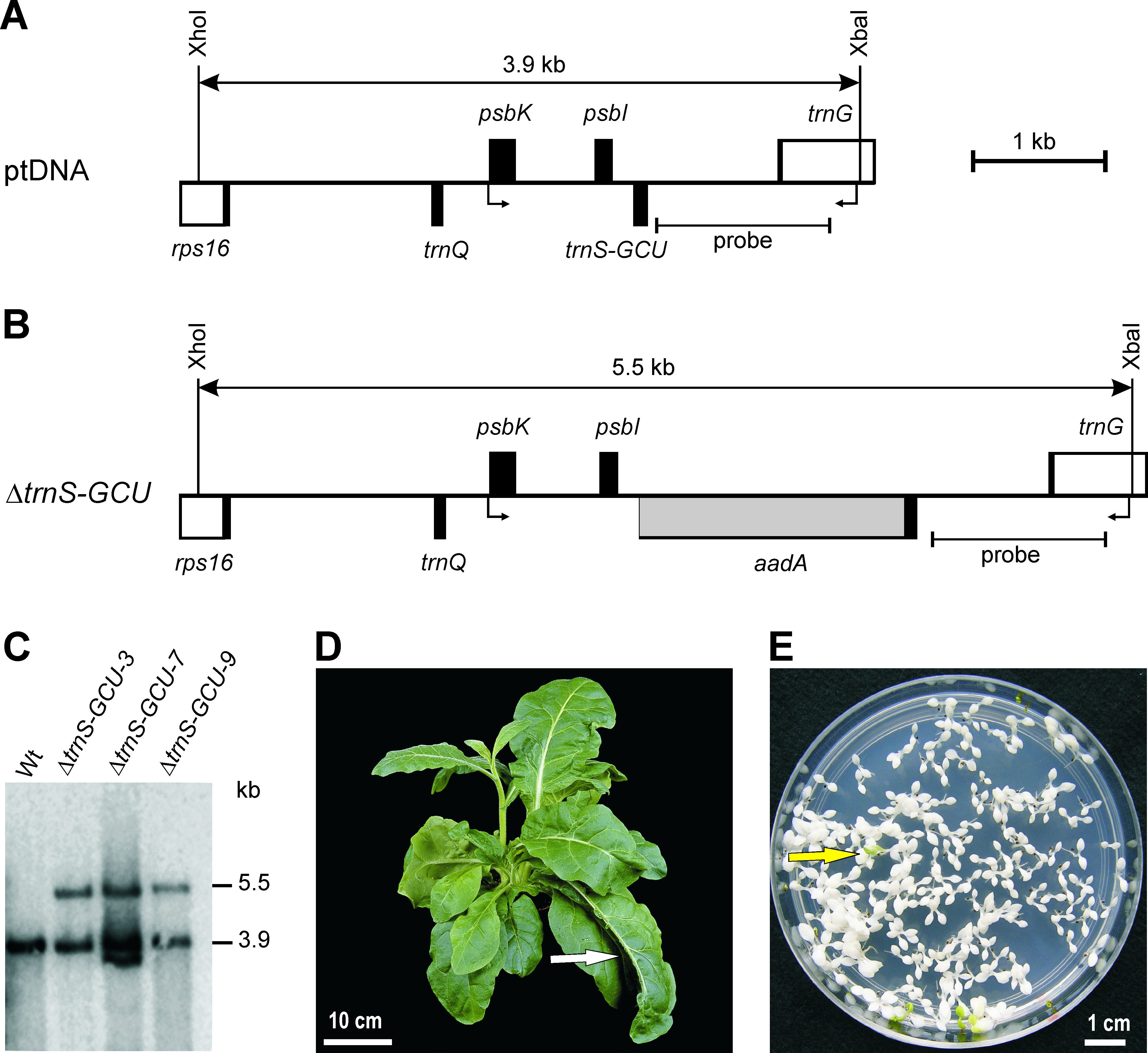

Supplement: Figure S3 — Targeted inactivation of the plastid trnS-GCU gene. (A) Physical map of the region in the tobacco plastid genome containing trnS-GCU. Genes above the line are transcribed from the left to the right, genes below the line are transcribed in the opposite direction. The bent arrows indicate the borders of the transformation vector. The restriction sites used for RFLP analysis are indicated. The hybridization probe and the expected size of detected DNA fragment are also shown. Introns are represented by open boxes. (B) Map of the transformed plastid genome obtained with plastid transformation vector pΔtrnS-GCU. The aadA selectable marker gene is shown in grey. (C) RFLP analysis of ΔtrnS-GCU plastid transformants. The transplastomic lines remain heteroplasmic and show both the 3.9 kb hybridization band diagnostic of the wild-type plastid genome and the 5.5 kb band diagnostic of the transplastome. Wt: wild type. (D) Leaf-loss phenotype of a typical heteroplasmic ΔtrnS-GCU plant. A misshapen leave is indicated by the arrow. (E) Inheritance assay of a ΔtrnS-GCU plant. An example of a spectinomycin-resistance seedling that has retained copies of the transplastome is indicated by the arrow. (TIF) [file pgen.1003076.s003.tif]

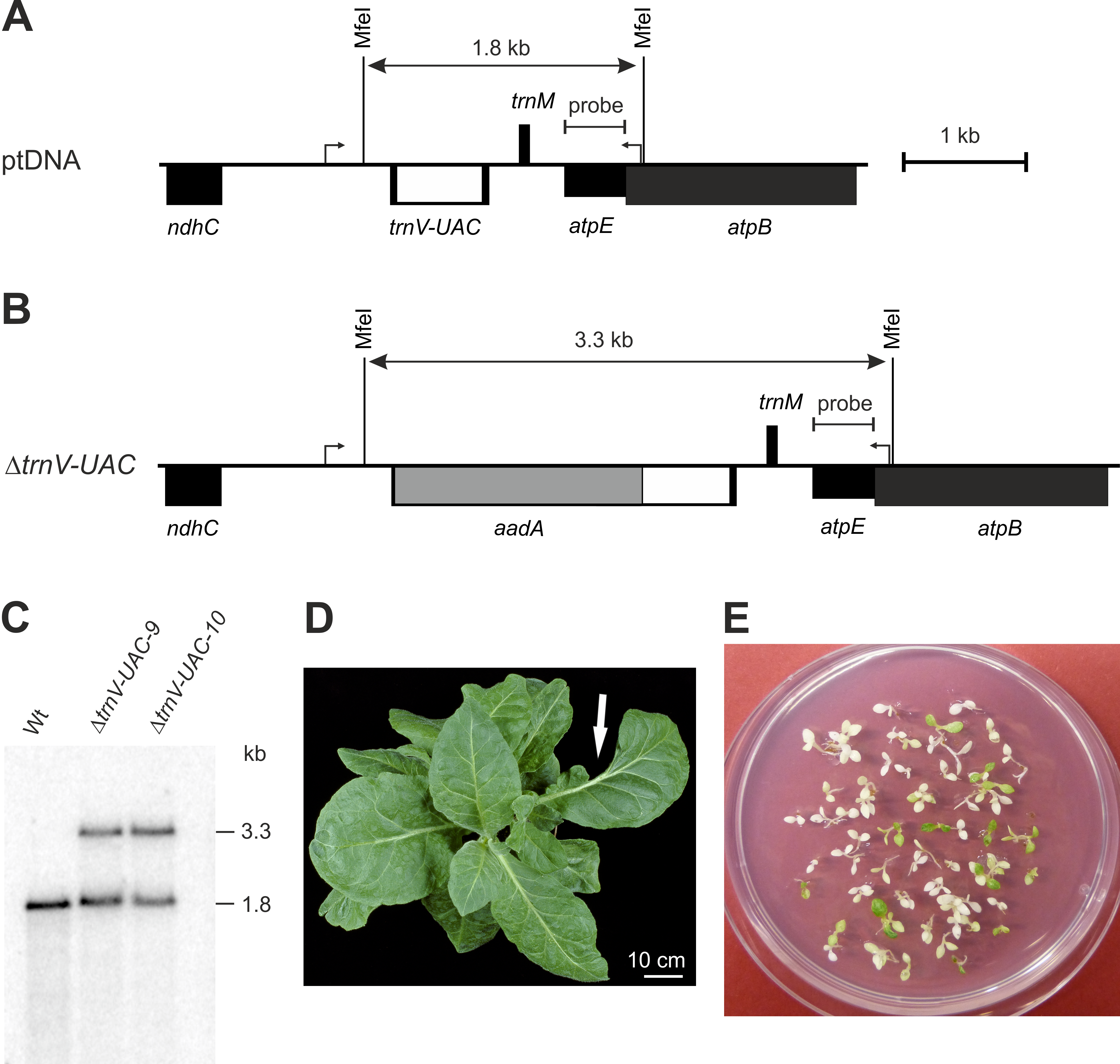

Supplement: Figure S4 — Targeted inactivation of the plastid trnV-UAC gene. (A) Physical map of the region in the tobacco plastid genome containing trnV-UAC. Genes above the line are transcribed from the left to the right, genes below the line are transcribed in the opposite direction. The bent arrows indicate the borders of the transformation plasmid. The restriction sites used for RFLP analysis are indicated. The hybridization probe and the expected size of the detected DNA fragment are also shown. The introns in trnV-UAC is represented by an open box. (B) Map of the transformed plastid genome (transplastome) produced with plastid transformation vector pΔtrnV-UAC. The aadA marker is shown as grey box. (C) RFLP analysis of ΔtrnV-UAC plastid transformants. The transplastomic lines remain heteroplasmic and show a stable ratio of the wild type-specific 1.8 kb band and the 3.3 kb band diagnostic of the transplastome. Wt: wild type. (D) Phenotype of a typical heteroplasmic ΔtrnV-UAC plant. The arrow points to an example of a misshapen leaf that lacks part of the leaf blade. (E) Inheritance assay of a ΔtrnV-UAC plant. Spectinomycin-resistance seedling that have retained copies of the transplastome are green, seedlings that have lost all copies of the transgenic plastid genome are white. (TIF) [file pgen.1003076.s004.tif]

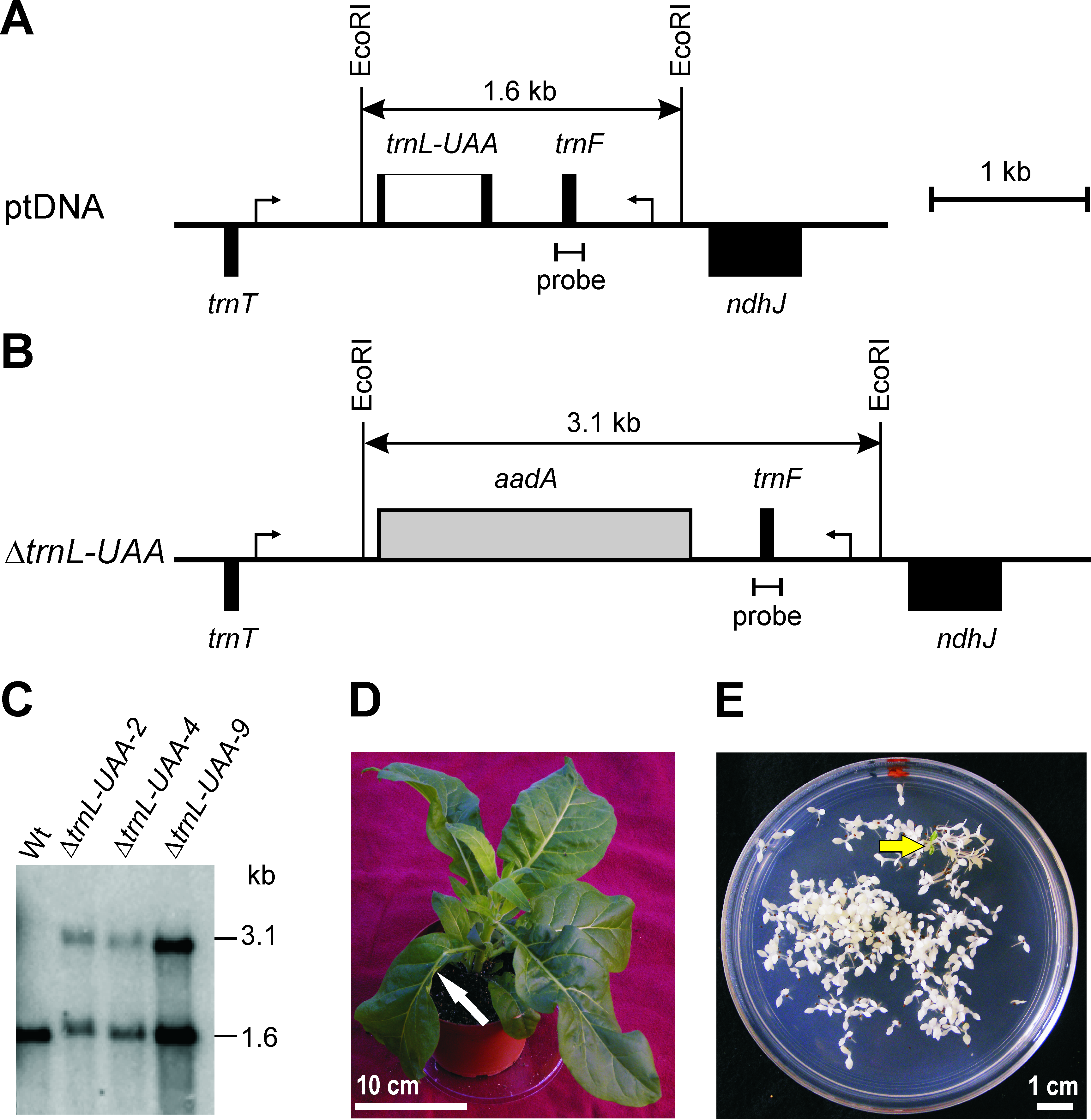

Supplement: Figure S5 — Targeted inactivation of the plastid trnL-UAA gene. (A) Physical map of the region in the tobacco plastid genome (ptDNA) containing the trnL-UAA gene. Genes above the line are transcribed from the left to the right, genes below the line are transcribed in the opposite direction. The bent arrows indicate the borders of the transformation plasmid. Restriction sites used for RFLP analysis are indicated. The hybridization probe and the expected size of the detected DNA fragment are also shown. The intron in the trnL-UAA gene is represented by an open box. (B) Map of the transformed plastid genome (transplastome) produced with plastid transformation vector pΔtrnL-UAA. The aadA cassette is shown as grey box. (C) RFLP analysis of ΔtrnL-UAA plastid transformants. The transplastomic lines remain heteroplasmic and show a stable ratio of the 1.6 kb hybridizing fragment diagnostic of the wild-type ptDNA and the 3.1 kb band diagnostic of the transformed ptDNA. Wt: wild type. (D) Phenotype of a typical heteroplasmic ΔtrnL-UAA plant. The arrow points to an example of a misshapen leaf. (E) Segregation analysis of a ΔtrnL-UAA plant. Seeds from a selfed transplastomic plant were sown on spectinomycin-containing synthetic medium. Spectinomycin sensitivity of most seedlings indicates that the plants tend to rapidly lose the transplastome in the absence of antibiotic selection. An example of a green spectinomycin-resistant seedling (that has retained the transplastome) is indicated by the arrow. (TIF) [file pgen.1003076.s005.tif]

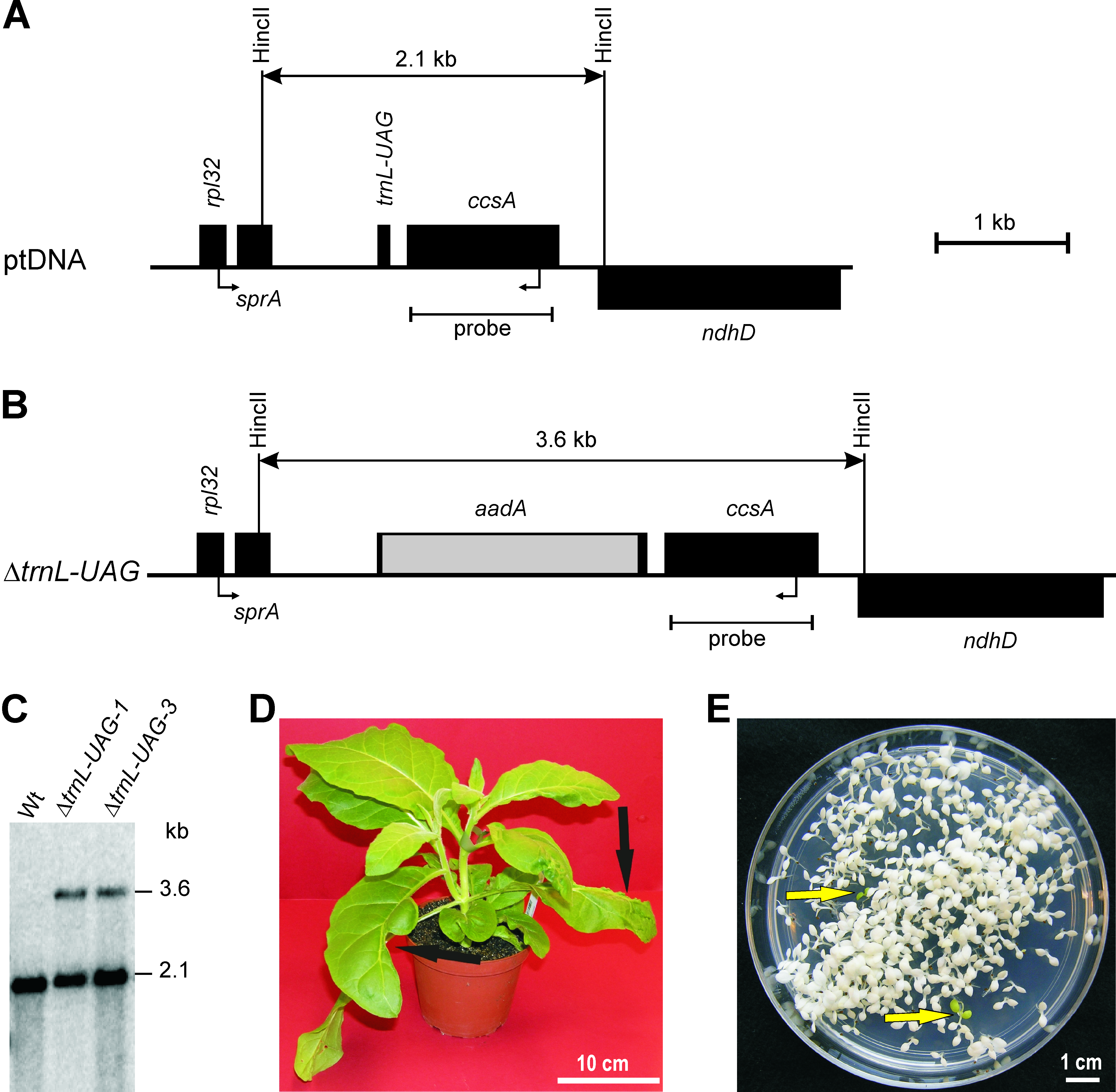

Supplement: Figure S6 — Targeted disruption of the plastid trnL-UAG gene. (A) Physical map of the region in the tobacco plastid genome containing the trnL-UAG gene. Genes above the line are transcribed from the left to the right, genes below the line are transcribed in the opposite direction. The bent arrows indicate the borders of the transformation plasmid. Restriction sites used for RFLP analysis are indicated. The hybridization probe and the expected size of detected DNA fragment are also shown. (B) Map of the transformed plastid genome obtained with plastid transformation vector pΔtrnL-UAG. The aadA selectable marker cassette is shown as grey box. (C) RFLP analysis of ΔtrnL-UAG chloroplast transformants. The transplastomic lines remain heteroplasmic and show both the wild type-specific 2.1 kb band and the transplastome-specific 3.6 kb band. Wt: wild type. (D) Phenotype of a typical heteroplasmic ΔtrnL-UAG plant. The arrow points to an example of a misshapen leaf that lacks part of the leaf blade. (E) Example of a seed assay confirming heteroplasmy of ΔtrnL-UAG plants and gradual loss of the transplastome in the absence of antibiotic selection. The transplastome is lost from most seedlings as evidenced by their white phenotype upon germination on spectinomycin-containing synthetic medium. The arrows points to two green (spectinomycin-resistant) seedlings that still harbor copies of the transplastome. (TIF) [file pgen.1003076.s006.tif]

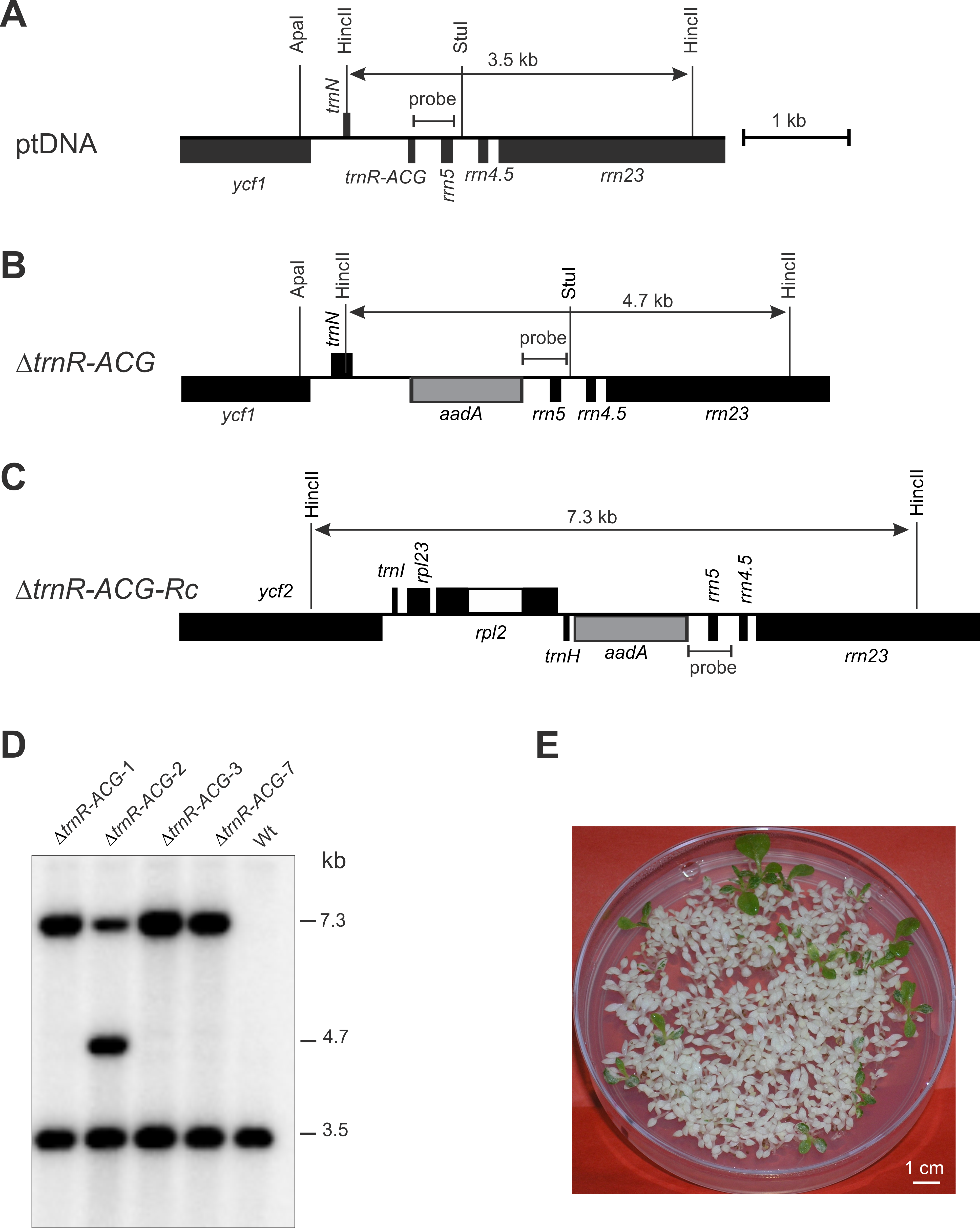

Supplement: Figure S7 — Targeted disruption of the plastid trnR-ACG gene. (A) Physical map of the region in the tobacco plastid genome containing the trnR-ACG gene. Genes above the line are transcribed from the left to the right, genes below the line are transcribed in the opposite direction. Selected restriction sites used for cloning and RFLP analysis are indicated. The hybridization probe and the expected sizes of detected DNA fragments are also shown. Introns are represented by open boxes. (B) Map of the transformed plastid genome (transplastome) produced with plastid transformation vector pΔtrnR-ACG. The aadA cassetten is shown as grey box. (C) Map of the recombination product (ΔtrnR-ACG-Rc) between the 3′ UTR the aadA cassette and that of the endogenous psbA gene. (D) RFLP analysis of ΔtrnR-ACG plastid transformants. All transplastomic lines remain heteroplasmic and show both the 3.5 kb wild type-specific hybridization band and a band diagnostic of the transplastome. The 7.3 kb band appearing in addition to or instead of the expected 4.7 kb transplastomic fragment in the transformants is the result of flip-flop recombination between the 3′ UTR of the aadA and that of the endogenous psbA gene [19]. Wt: wild type. (E) Inheritance assay of a ΔtrnR-ACG plant. Spectinomycin-resistance seedlings that have retained the transplastome are green on antibiotic-containing medium. The phenotype of heteroplasmatic ΔtrnR-ACG lines is shown in Figure S8E. (TIF) [file pgen.1003076.s007.tif]

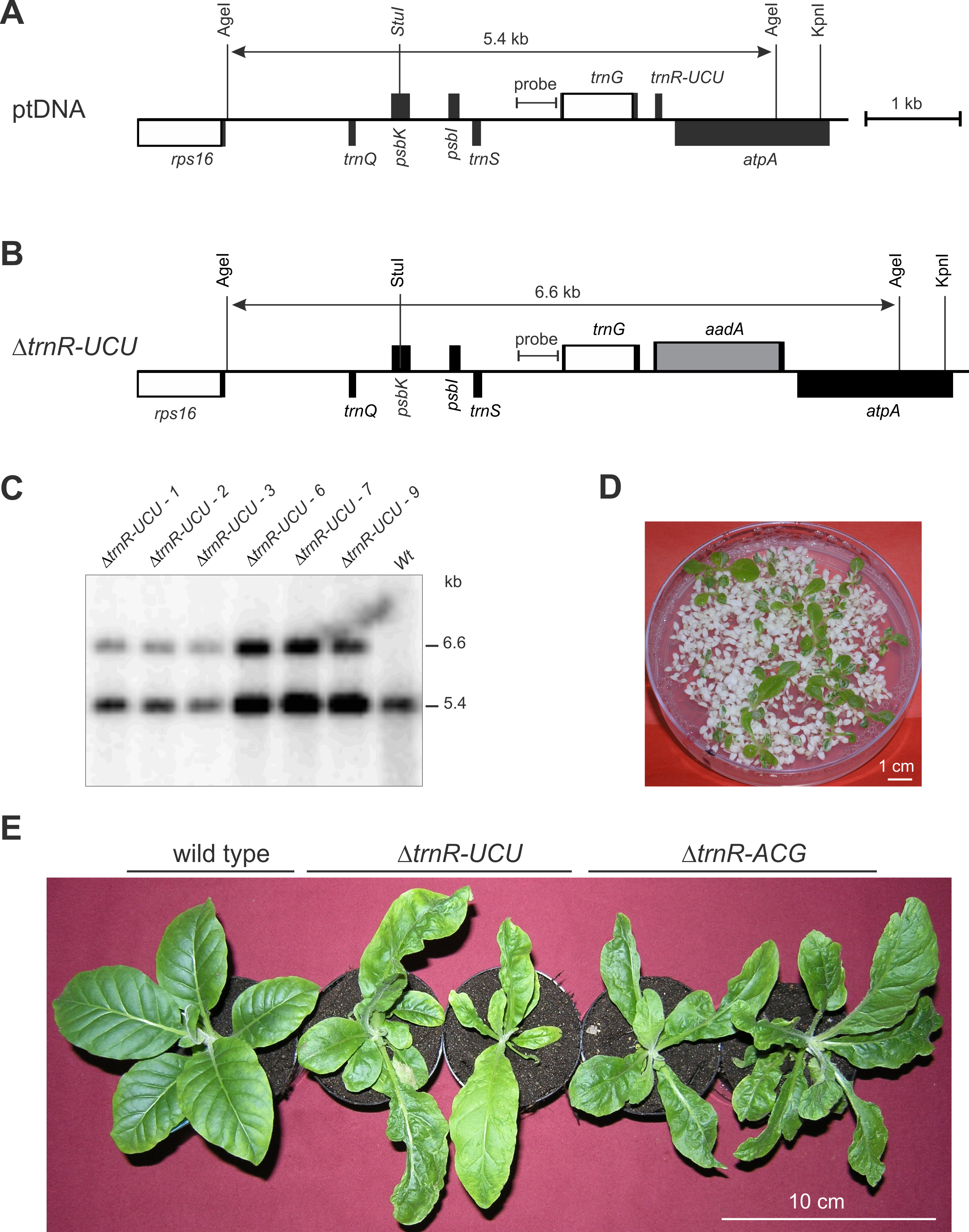

Supplement: Figure S8 — Targeted disruption of the plastid trnR-UCU gene. (A) Physical map of the region in the tobacco plastid genome containing the trnR-UCU gene. Genes above the line are transcribed from the left to the right, genes below the line are transcribed in the opposite direction. Selected restriction sites used for cloning and RFLP analysis are indicated. The hybridization probe and the expected size of detected DNA fragments are also shown. Introns are represented by open boxes. (B) Map of the transformed plastid genome (transplastome) produced with plastid transformation vector pΔtrnR-UCU. The aadA marker cassette is shown as grey box. (C) RFLP analysis of ΔtrnR-UCU plastid transformants. The transplastomic lines remain heteroplasmic and show both the 5.4 kb wild type-specific hybridization band and the 6.6 kb band diagnostic of the transplastome. Wt: wild type. (D) Inheritance assay of a ΔtrnR-UCU plant. Spectinomycin-resistance seedlings that have retained the transplastome are green on antibiotic-containing medium. (E) Phenotype of heteroplasmic ΔtrnR-UCU and ΔtrnR-ACG plants. A wild-type plant (left), two ΔtrnR-UCU plants (middle) and two ΔtrnR-ACG plants grown under greenhouse conditions are shown. Misshapen leaves with missing sectors indicate essentiality of the trnR-UCU and ΔtrnR-ACG genes. (TIF) [file pgen.1003076.s008.tif]

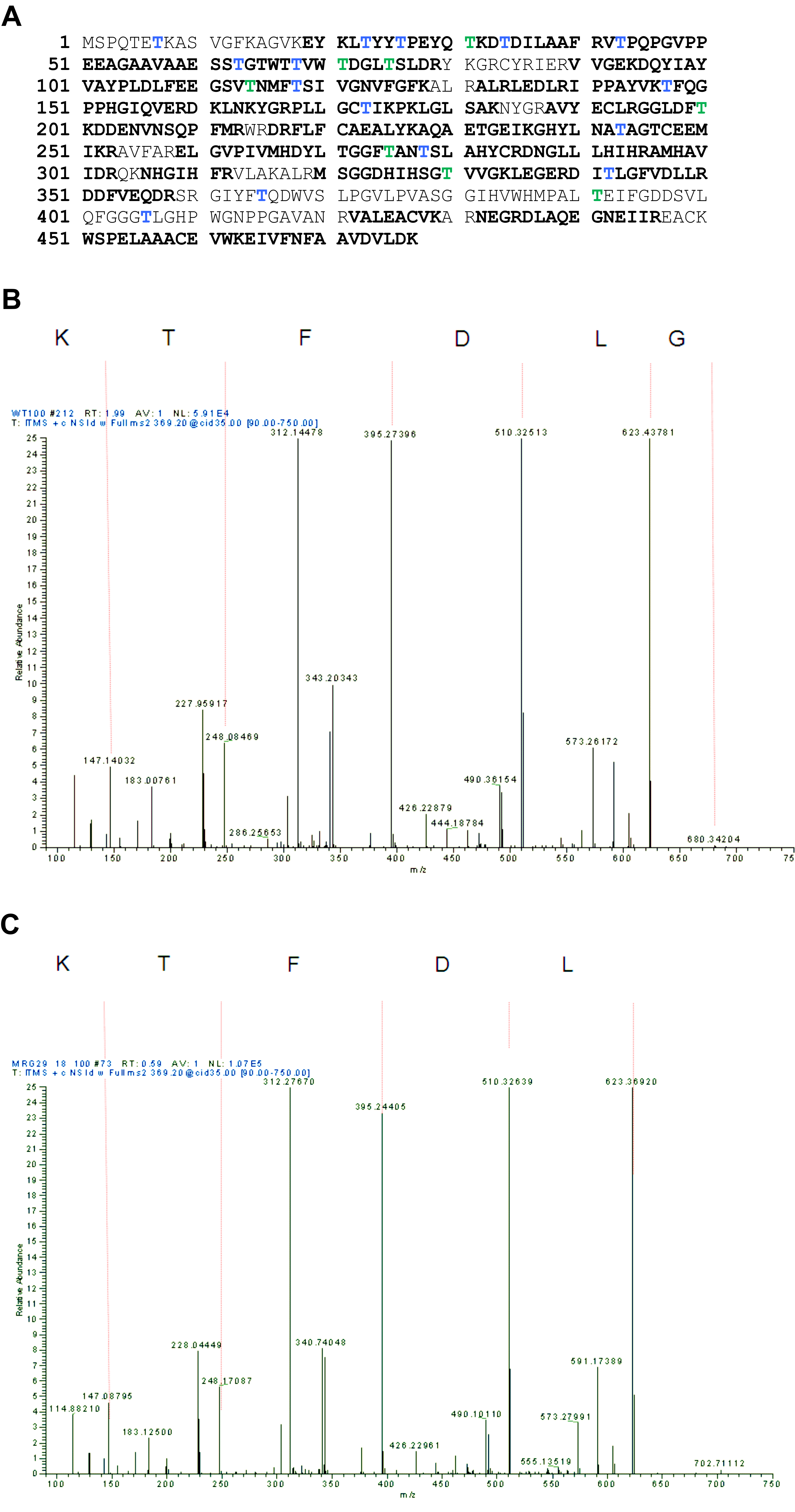

Supplement: Figure S9 — Verification of threonine incorporation into the large subunit of Rubisco (RbcL) in the ΔtrnT-GGU plants by MS/MS. (A) Detected RbcL peptides and threonine residues dependent on superwobbling in the ΔtrnT-GGU mutant. The RbcL peptides detected by mass spectrometry are marked in bold. The threonines encoded by ACU and ACC codons are indicated in blue and green, respectively. These codons are decoded by tRNA-Thr(GGU) in the wild type, but are read by tRNA-Thr(UGU) in the ΔtrnT-GGU transplastomic lines using superwobbling. In all detected peptides, threonine was correctly incorporated. (B) Confirmation of threonine incorporation into RbcL by de novo sequencing. Example of a y-ion series from a peptide containing an ACC-encoded threonine from the wild-type sample. (C) Example of a y-ion series from a peptide containing an ACC-encoded threonine from a ΔtrnT-GGU plant. (TIF) [file pgen.1003076.s009.tif]
