# Supplementary material for: The Contributions of Wobbling and Superwobbling to the Reading of the Genetic Code
Source: PLoS Genet. 2012 Nov 15;8(11):e1003076. doi: 10.1371/journal.pgen.1003076 (PMC3499367; doi:10.1371/journal.pgen.1003076)
Supplement: Table S1 — Numbers of codons in the plastid genome affected by the knock-out of the four non-essential tRNA genes trnL-CAA, trnS-GGA, trnT-GGU and trnV-GAC. The total numbers of codons for leucine, serine, threonine and valine in all plastid protein-coding genes are given as well as the numbers of codons that are optimally decoded by each of the knocked-out tRNA species. (DOC) [file pgen.1003076.s010.doc]

**Table S1. Numbers of codons in the plastid genome affected by the knock-out of the four non-essential tRNA genes *trnL-CAA*, *trnS-GGA, trnT-GGU* and *trnV-GAC*.** The total numbers of codons for leucine, serine, threonine and valine in all plastid protein-coding genes are given as well as the numbers of codons that are optimally decoded by each of the knocked-out tRNA species.

| **Deleted tRNA gene** | *trnL-CAA* | *trnS-GGA* | *trnT-GGU* | *trnV-GAC* |
| --- | --- | --- | --- | --- |
| **Number of codons** | 2469 | 1697 | 1167 | 1283 |
| **Affected codons** | 493 | 766 | 688 | 654 |
