# Supplementary material for: The Contributions of Wobbling and Superwobbling to the Reading of the Genetic Code
Source: PLoS Genet. 2012 Nov 15;8(11):e1003076. doi: 10.1371/journal.pgen.1003076 (PMC3499367; doi:10.1371/journal.pgen.1003076)
Supplement: Table S2 — Sequences of oligonucleotides used for cloning and/or generation of hybridization probes. Sequences complementary to sequences in the aadA cassette are underlined. (DOC) [file pgen.1003076.s011.doc]

**Table S2. Sequences of oligonucleotides used for cloning and/or generation of hybridization probes.** Sequences complementary to sequences in the aadA cassette are underlined.

| **Primer** | **Sequence (5’ → 3’)** |
| --- | --- |
| P5_trnS | GCGATCCTCTTTCCGTACCTTCGC |
| P3_ Bsu36ItrnS | GGCGAAGTAAATTAACCTAAGG |
| P5_sense | CCTAGGTCAGTTTATGGGCT |
| P3_ antisense | GTGCTTCAGGACCCCATAGTAA |
| P5_16SkomplettF | gagagttcgatcctggctca |
| P3_ 16SkomplettR | GCACCTTCCAGTACGGCTAC |
| P5_atpEprfor | TTGATTGTATTCACCCGACAGAATC |
| P3_atpEprrev | TGGGATTCAGAAGTGGAAGAAATTG |
| P5_trnLUAGFRAGFOR | TCGCATTAAAGGCTTTTTCA |
| P3_trnLUAGFRAGREV | GGTCCCAATTCCAATACGAC |
| P5_trnLUAAmod1 | tgactaaactattggaattggaatct |
| P3_trnLUAAmod2 | aaattcggttgaacgagagg |
| P3_trnLUAAmod3 | CTCACTTTTCTTGGATCctttctttttgagattaggatctc |
| P5_trnLUAAmod4 | gagctcggtacccgggaaaagactatttcactcccca |
| P5_trnLUAAaadAkass5 | tcaaaaagaaagGATCCAAGAAAAGTGAGCTATT |
| P3_trnLUAAaadAkass6 | gtgaaatagtcttttcccgggtaccgag |
| P5_ 5’ClaItrnS | GGAATCGATAAGAAATAAGAAATATTTC |
| P3_ 3’trnS | CACTTTTCTTGGATCTACATAACGATTATGACCCAAAAAC |
| P5_5’aadAtrnS | CGTTATGTAGATCCAAGAAAAGTGAGCTATTAAC |
| P3_3’aadAtrnS | GTACGGAAAGAGGATCGCACTCTACCGA |
| P5_5’trnS | GCGATCCTCTTTCCGTACCTTCGC |
| P3_ 3’Bsu36ItrnS | GGCGAAGTAAATTAACCTAAGG |
| P5_trnVfor | CATAGGTTAGGTACAGTGTTTG |
| P3_trnVrev | GCCTAGTATCCATCGTTTAC |
| P5_trnV-GAC-aadA-hin | GGGATCCAAGAAAAGTGAGCTATTAAC |
| P3_trnV-GAC-aadA-rev | CATTGGGCTTTTAGTATGTTACTATTTCTTTTATTATAACTTATAAAATATAATAC |
| P5_trnV-GAC-MfeI-hin | GAGGTAAATCTTTGCCTGCTAG |
| P3_trnV-GAC-MfeI-rev | TCACTTTTCTTGGATCCCGTCCCCGCCCCATCG |
| P5_ trnV-GAC-BbvCI-hin | GAAATAGTAACATACTAAAAGCCCAATGTGAGTTTTTCTAG |
| P3_ trnV-GAC-BbvCI-rev | AGTTTCCACCGCCTGTC |
| P5_trnV-UAC-for | tcttcgtagtttttcatttcacca |
| P3_trnV-UAC-rev | gcagaaacaattcgaggatttc |
| P_trnL-CAA-for | tgccttgaagaggactcg |
| P_trnL-CAA-rev | gccttggtggtgaaatggtag |
| P_trnS-GGA | aacaaaagcctacatagcagttccaatgctacgccttcaa |
| P_trnV-GAC-for | tctatttctcgatggggc |
| P_trnV-GAC-rev | tagggataatcaggctcg |
| P_trnT-GGU-A | TGTACCTATAGATATTTTATCCAC |
| P_trnT-GGU-B | TAAAAATACAATTCTCCCCTCAAA |
